# Proteus: An algorithm for proposing stabilizing mutation pairs based on interactions observed in known protein 3D structures

**DOI:** 10.1186/s12859-020-03575-6

**Published:** 2020-07-01

**Authors:** José Renato M. S. Barroso, Diego Mariano, Sandro R. Dias, Rafael E. O. Rocha, Lucianna H. Santos, Ronaldo A. P. Nagem, Raquel C. de Melo-Minardi

**Affiliations:** 1 Department of Computer Science, Laboratory of Bioinformatics and Systems, Universidade Federal de Minas Gerais, Belo Horizonte, 31270-901 Brazil; 2 Departament of Computing, CEFET-MG, Belo Horizonte, 30510-000 Brazil; 3 Department of Biochemistry and Immunology, Universidade Federal de Minas Gerais, Belo Horizonte, 31270-901 Brazil

**Keywords:** Protein engineering, Mutations, Webtool, Algorithm, Database

## Abstract

**Background:**

Protein engineering has many applications for industry, such as the development of new drugs, vaccines, treatment therapies, food, and biofuel production. A common way to engineer a protein is to perform mutations in functionally essential residues to optimize their function. However, the discovery of beneficial mutations for proteins is a complex task, with a time-consuming and high cost for experimental validation. Hence, computational approaches have been used to propose new insights for experiments narrowing the search space and reducing the costs.

**Results:**

In this study, we developed Proteus (an acronym for Protein Engineering Supporter), a new algorithm for proposing mutation pairs in a target 3D structure. These suggestions are based on contacts observed in other known structures from Protein Data Bank (PDB). Proteus’ basic assumption is that if a non-interacting pair of amino acid residues in the target structure is exchanged to an interacting pair, this could enhance protein stability. This trade is only allowed if the main-chain conformation of the residues involved in the contact is conserved. Furthermore, no steric impediment is expected between the proposed mutations and the surrounding protein atoms. To evaluate Proteus, we performed two case studies with proteins of industrial interests. In the first case study, we evaluated if the mutations suggested by Proteus for four protein structures enhance the number of inter-residue contacts. Our results suggest that most mutations proposed by Proteus increase the number of interactions into the protein. In the second case study, we used Proteus to suggest mutations for a lysozyme protein. Then, we compared Proteus’ outcomes to mutations with available experimental evidence reported in the ProTherm database. Four mutations, in which our results agree with the experimental data, were found. This could be initial evidence that changes in the side-chain of some residues do not cause disturbances that harm protein structure stability.

**Conclusion:**

We believe that Proteus could be used combined with other methods to give new insights into the rational development of engineered proteins. Proteus user-friendly web-based tool is available at <http://proteus.dcc.ufmg.br>.

## Background

The design of new proteins, known as protein engineering, has many applications for industry. Novel and likely enhanced proteins can benefit the development of new drugs, vaccines, treatment therapies, and improve the enzymes used in digestive processes for new food and biofuel production [1–4]. Industrial biotechnological applications require enzymes with higher thermal and conformational stability [5]. Hence, the study of the effect of mutations in proteins may help better understand the forces that stabilize macromolecules, allowing modifications in protein properties [6]. However, probing mutations by experimental assays is a complex, time-consuming, and high-cost task. Computational approaches have been used to propose insights for experiments and help improve enzymes, which may reduce costs, time of development and increase the rational design of enzymes [6].

Since in recent years, a considerable number of sequences from different organisms has been available mainly due to the evolution of next-generation sequencing technologies [7]. Sequence-based strategies rely on evolutionary conservation to predict mutation impacts. Examples such as MuStab [8] and iPTREE-STAB [9] have been implemented to predict protein stability changes upon amino acid substitutions based on sequences. However, most sequence-based approaches do not consider structural information, which is closely related to the protein function. SDM, a structure-based approach, uses substitution tables to analyze if replaced amino acids are tolerated within the family of homologous proteins [10]. Also, graph-based signatures and machine learning have been used to predict stabilizing mutations for proteins [11].

Although many strategies have been proposed, the prediction of stabilizing mutations in target proteins through computational methods is still an open problem. This occurs due to the large number of possible mutations. In addition, even the in silico prediction of the impact caused by switching more than one amino acid is a complex task. Therefore, we hypothesized that using known structures is a better path to suggest new stabilizing mutations.

In this paper, we present Proteus, a web server that proposes mutated residue pairs observed in known structures for improving the stability of a target protein. We performed two case studies to illustrate Proteus’ potential for suggesting mutations for enzymes of industrial applications. Furthermore, we performed qualitative comparisons among Proteus and several other tools. Since Proteus introduces a new algorithm for suggesting mutations, it is not possible to perform a direct numerical comparison between methods. However, we believe that Proteus could be used together with these other tools to better predict promising mutations for rational protein design.

### Related works

Several methods, tools, and web servers have been developed to propose mutations in target proteins (Supplementary Table S1). The first computational strategies aimed to insert new disulfide bonds in macromolecules to improve their thermostability. For example, SSBOUND [12] and MODIP software [13] use a classification system based on conformation parameters for disulfide bonds (distances of 1.87 Å and 2.04 Å for Cβ-S and S-S, respectively; and angles of 114° and 104° for CαCβS and CβSS, respectively) to suggest locations where the introduction of a disulfide cross-link could lead to protein stabilization.

With the advent of the new sequencing technologies, an abundant number of primary structures from several proteins in different organisms allowed the emergence of new sequence-based strategies for the prediction of beneficial mutations in target proteins. For instance, MuStab is a sequence-based tool that uses machine learning to predict protein stability changes upon amino acid substitutions [8]. Another example is the iPTREE-STAB web server that aims to predict protein stability changes upon single amino acid substitutions [9].

Structure-based approaches have also been used to predict mutation impacts. PopMuSiC webtool predicts thermodynamic stability changes using a linear combination of statistical potentials based on the solvent accessibility of the mutated residue [5]. SDM web server uses substitution probability tables obtained from known 3-D structures to analyze the variation of amino acid replacements tolerated within the family of homologous proteins [10]. mCSM uses graph-based signatures and pharmacophore properties in a machine learning approach to predict stabilizing and destabilizing mutations [11]. Also, SDM and mCSM methods were combined in a hybrid approach called DUET, which tries to obtain a more accurate prediction of the free energy change; ΔΔG [14]. SSV is another method that uses graph-based structural signatures and compares Euclidean distances between vectors to predict if mutations introduce similar characteristics to reference proteins [15]. Lastly, MAESTRO is a web server that combines high-throughput scanning for multi-point mutations, prediction of free energy change, and stabilizing disulfide bonds [6]. To the best of our knowledge, no tool to suggest amino acid pair substitutions that insert new stabilizing contacts based on conformations observed on known 3D structures were found.

## Results

**Fig. 1:**
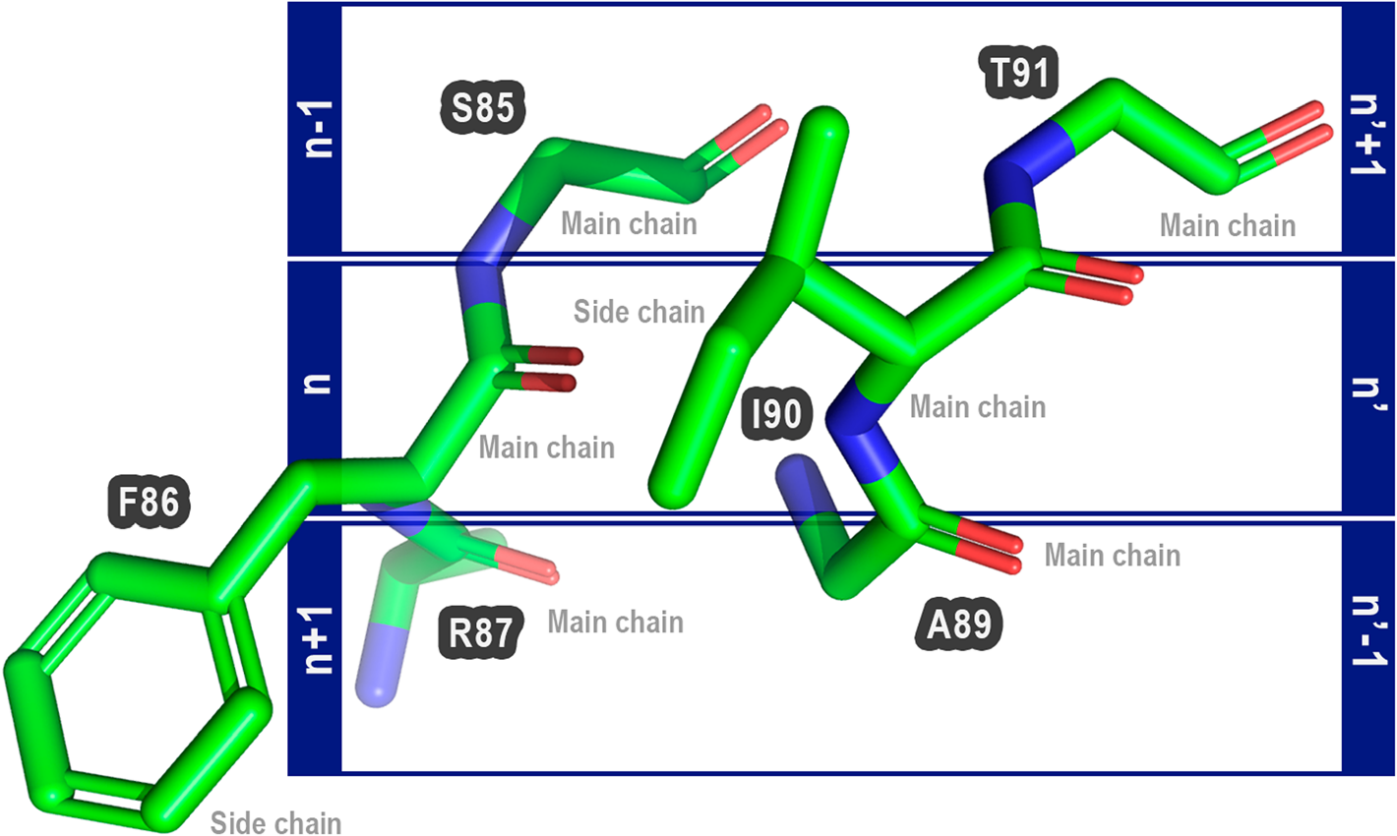
Composition of triad pairs. Amino acids that could make interactions are called n and n’. The anterior and posterior are called n-1 and n + 1 for n, and n’-1 and n’ + 1 for n’. In this example, the amino acid F86 (PDB ID: 1LGY:A) is not performing interactions with I90 (PDB ID: 1LGY:A), but they are in a cutoff distance position that could allow interactions if they were changed. The anterior and posterior are S85 and R87 (F86), and A89 and T91 (I90). The Proteus’ basic assumption is that is possible to trade the amino acids n and n’ to another amino acid pair if the main chain of triad pairs of target and template protein were conserved

Proteus (an acronym for Protein Engineering Supporter) is based on the assumption that if a pair of non-interacting amino acid residues are changed to a pair of interacting amino acids, this could improve protein stability. Furthermore, these mutations would only be allowed if the main-chain conformation of two sets of three amino acids (herein called triad pairs) were conserved between the target and the proposed mutations. These triad pairs are composed of the amino acid residues in close contact, and the previous and posterior residues. Amino acids that could be mutated are called in our notation n and n’ (Fig. 1).

We hypothesized that if the main-chain conformation of triad pairs is not changed upon mutations, i.e.*,* if the proposed mutations introduce a pair of interacting residues without modifying the main chain trace of the native triad pairs; then, the substitution would be allowed. We believe that this is possible because of this interaction is a real and possible conformation already observed for a specific pair of interacting residues. Thus, the interaction might occur in the mutated protein and could improve its stability when compared to the wild macromolecule.

Aiming to evaluate this strategy, we developed Proteus, a structure-based algorithm accessible as a web-based tool. The software receives as input a protein 3D target, and then it selects every two amino acids in close distance to each other (not in direct contact) and their four neighboring residues (a pair for possible mutations within the triad pair). A comparison between each selected triad pairs in the target protein and the triad pairs in Proteus Data Bank (also called ProteusDB; main-chain conformation comparison) allows the identification of potential mutation pairs that could be introduced into the target protein, improving its stability without significant conformational changes. This is possible because each triad pairs in ProteusDB is formed by two interacting residues (n and n’) and their respective neighboring residues, as collected from all available structures at the PDB (Protein Data Bank, available at http://www.rcsb.org/pdb).

### Proteus software suite

**Fig. 2:**
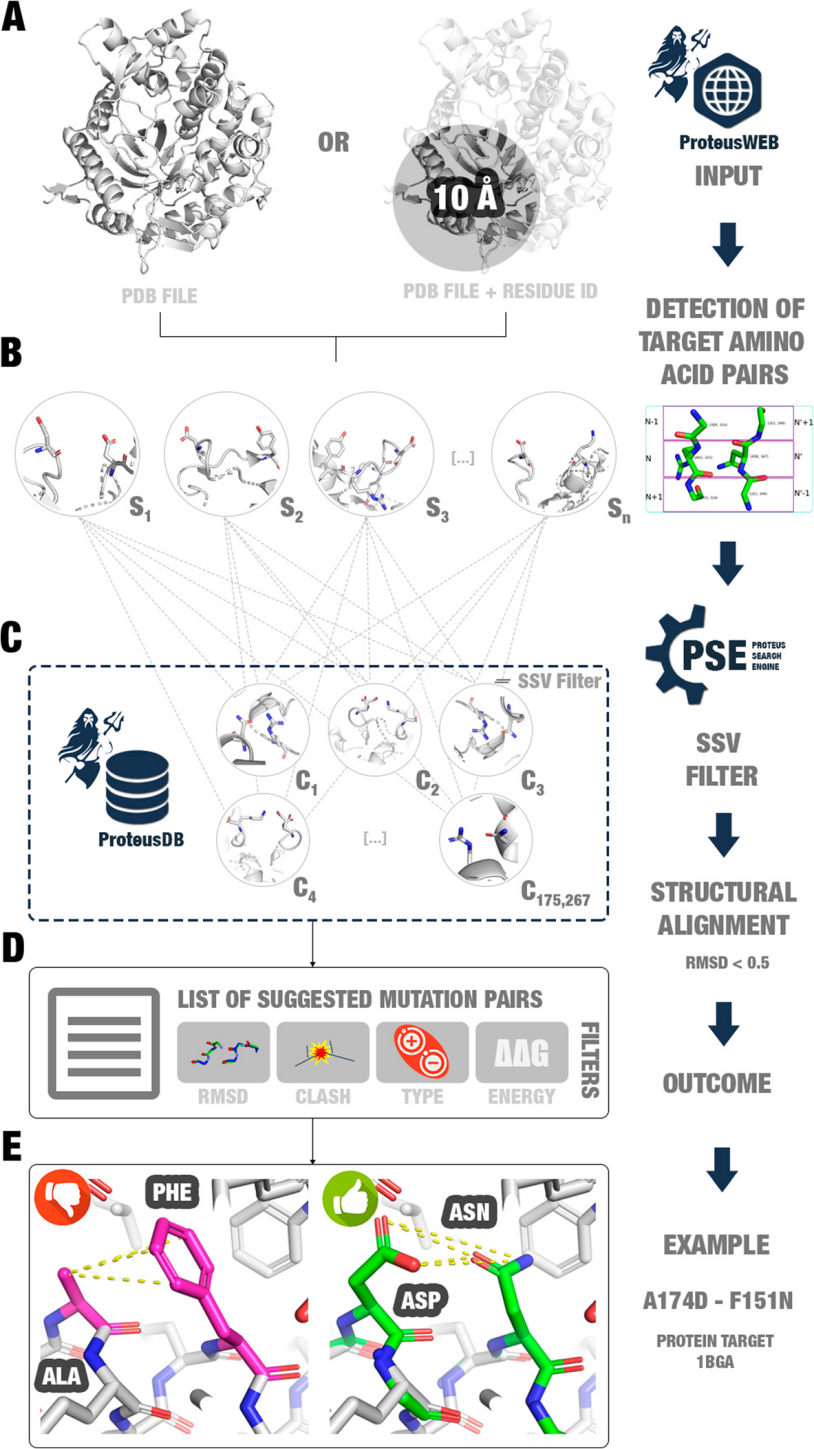
Proteus’ workflow. **a** ProteusWEB receives as input a PDB file or a region to search for possible beneficial mutations. **b** ProteusWEB detects target amino acid pairs (alpha carbons at a distance between 3.35–16.40 Å). **c** Each triad pair containing the target pair is aligned against ProteusDB triad pairs. Only main chain structural alignments between triad pairs from the target and ProteusDB with RMSD < 0.5 Å are selected. **d** ProteusWEB returns a list of suggested mutation pairs, which can be filtered by users. **e** Example of a suggested mutation pair for a β-glucosidase enzyme (PDB: 1BGA)

Proteus assumes that if two amino acid residues, in close but not in direct contact with each other, are simultaneously mutated to another pair of interacting residues, this could improve protein stability. This residue exchange is proposed from known 3D-structures (derived from PDB) that preserves not only the same interactions between the target chosen pair but also the main-chain conformation. The main chain that needs to be maintained is composed of two sets of three amino acid residues (triad pairs). Summarizing, the triad pairs contain two interacting residues (n and n’, in our notation), two preceding (n-1 and n’-1), and two subsequent (n + 1 and n’ + 1) residues (Fig. 2). We suppose that no steric impediment is allowed between the proposed mutations and the surrounding protein atoms. Proteus software suite is divided into three main parts:

(i) **ProteusWEB**: a user-friendly web tool interface, which receives as input a PDB file. Then, Proteus selects possible pairs of target amino acids for substitution and their adjacent residues (main chain triad pairs). Each triad pair is compared to the ProteusDB using the PSE (Proteus Search Engine) method. ProteusWEB returns a list of possible stabilizing mutations, that could be evaluated by several filters, such as RMSD between target and template triad pairs, clash verification, the type of amino acids in contact, and prediction of mutation impact using ΔΔG calculated by the MAESTRO software [6]. Also, Proteus allows the visualization of the structures using 3Dmol [16].
(ii) **ProteusDB**: a database of interactions composed of 175,267 three-dimensional structures of the main chain of six amino acids (n-1, n, n + 1, n’-1, n’, and n’ + 1) and the side-chains of two residues (n and n’). The interactions found in ProteusDB are hydrogen bonds, ionic interactions, and disulfide bonds. The data were collected from the PDB and clustered to represent all possible conformations for each specific interaction.
(iii) **PSE (Proteus Search Engine)**: the search method used for discovering possible beneficial mutations. PSE uses Biopython [17] for structural alignment between the target and ProteusDB triad pairs. If the RMSD is lower than 0.5 Å, PSE considers that both residues could be exchanged simultaneously without undesirable main chain conformational changes in the structure. Furthermore, PSE uses SSV (structural signature variation) method [15] to reduce computational costs (see implementation section for details).

### Proteus web-based tool

**Fig. 3:**
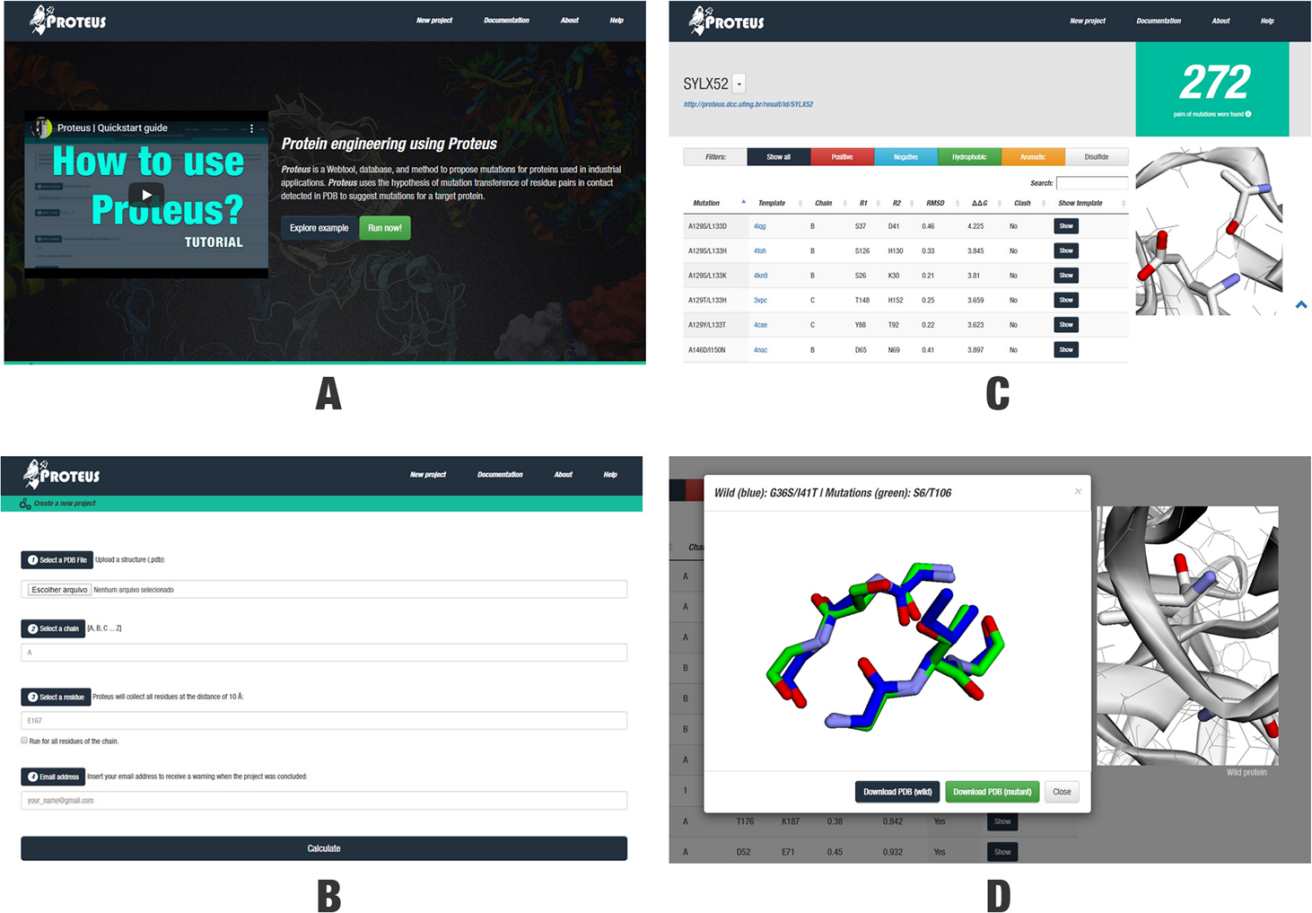
**a** Proteus’ interface. **b** Four input parameters are required as input: (i) a PDB file (mandatory); (ii) an amino acid chain (mandatory; character, e.g. A); (iii) a residue identifier (optional; for fast search; e.g. E167); and (iv) your email address. **c** Project’s page. At the top of the page, users can see the project’s name with a link to the page, and the number of mutation pairs suggested by the algorithm. Users can utilize a list of filters (“Show all”, “Positive”, “Negative”, “Hydrophobic”, “Aromatic”, and “Disulfide”) and routine parameters (“Mutation”, “Template”, “Chain”, “*n* = R1”, “n’ = R2”, RMSD, ΔΔG, and Clash) to sort the proposed mutations according to their own interests. On the right side, users can use the 3D-visualization window to analyze the three-dimensional structure of the target protein (zoom in, zoom out, rotate, and translate). If users want to have a closer view of a specific suggested mutation pair, they can click on the respective “Show” button to show a structural alignment modal. **d** The structural alignment modal presents the alignment between the triad pairs of wild and mutant (obtained from the template). On the right side, it is available the complete structure of the target protein

Proteus presents a user-friendly interface. The users can create a new project inserting: (i) a PDB file; (ii) the name of a target chain; (iii) a residue followed by its number position to perform a fast search in the region until 10 Å from this residue (Fig. 3a; optionally, the user can select a box that allows a search in the whole protein); and (iv) an email address to receive a message when the processing finishes. After processing, Proteus returns a list of mutations suggested summarized by mutation sites (Fig. 3b) or a complete list that could be sorted by several filters, such as amino acid properties, the structure where the mutation was observed, the RMSD, ΔΔG, and stereochemistry clash (Fig. 3c). Proteus indicates promising mutation pair substitutions for the target protein and allows to compare the structural alignment between wild and mutant triad pairs (Fig. 3d). We expected that mutations suggested based on our strategy present lower RMSD between triad pair main chains, lower Gibbs free energy variation (ΔΔG), and no stereochemistry clash between atoms when the substitutions were performed. However, in some cases nor all these characteristics are achieved. Hence, Proteus’ interface shows all results, allowing the user to take the decision of what mutation is more promising for experimental validation.

### Case studies

To evaluate Proteus, we performed two case studies. In the first one, we evaluated if suggested mutations attend to the basic hypothesis of Proteus: increasing the number of contacts among residues. Thus, we selected structures from PDB and submitted them to Proteus. We performed in silico mutations according to the Proteus algorithm and the coordinates established by the template structures. Then, we determined the total of contacts in wild and mutant structures using Arpeggio tool [18].

In the second case study, we collected a list of mutations experimentally validated from the ProTherm database [19–21]. Then, we ran Proteus predictions for a lysozyme protein with data available for several mutations validated experimentally. Lastly, we compared the predicted outcome with experimental data.

#### Case study 1: evaluating Proteus hypothesis

We collected four structures from PDB:

(i) ***Bacillus polymyxa*GH1 β-glucosidase (PDB ID: 1BGA):** β-glucosidases are vital enzymes in the second-generation biofuel production. They act cleaving cellobiose in two molecules of glucose, which will be used in fermentation for producing bioethanol [3]. The design of thermostable and more efficient β-glucosidases enzymes has excellent value for the industry [4];
(ii) **tobacco etch virus protease (PDB ID: 1LVB):** proteases (peptidases) hydrolyze the peptide bond producing single amino acids or smaller polypeptides molecules. They are essential for several biological functions in all forms of life. Hence, we have chosen a protease from the tobacco etch virus for this case study [22];
(iii) **immunoglobulin new antigen receptor (PDB ID: 2YWY):** we selected the structure of an antigen receptor variable domain from sharks for the third example of this case study [23];
(iv) **lipase (PDB ID: 1LGY)**: lastly, we selected the structure of the lipase II obtained from *Rhizopus niveus* [24].

These structures were submitted to Proteus for suggesting mutations (Table 1; Tables S2, S3, S4, S5). For the *Bacillus polymyxa* GH1 β-glucosidase (PDB ID: 1BGA), Proteus suggested 344 mutation pairs (Table 1). For the catalytically inactive tobacco etch virus protease (PDB ID: 1LVB), Proteus found 61 possible sites for mutations. In the third example, Proteus found 50 possible sites for mutations in the immunoglobulin new antigen receptor (PDB ID: 2YWY). Lastly, for lipase II from *Rhizopus niveus* (PDB ID: 1LGY) [24], Proteus found 213 possible sites for mutations (Table 1).

**Table 1:** Proteus’ results for the first case study (details were included in the supplementary material: Tables S2, S3, S4, S5). (1) β-glucosidase project is available at: http://proteus.dcc.ufmg.br/result/id/4L8HLU. (2) Protease project is available at: http://proteus.dcc.ufmg.br/result/id/N7Q9RZ. (3) Immunoglobulin new antigen receptor project is available at: http://proteus.dcc.ufmg.br/result/id/UM6SQD. (4) Lipase project is available at: http://proteus.dcc.ufmg.br/result/id/YTP3YC

#	Protein	PDB ID	ProteusID	Sequence length	Number of mutations suggested
1	β-glucosidase (hydrolase)	1BGA	4L8HLU	447	344
2	Protease	1LVB	N7Q9RZ	243	61
3	Immunoglobulin new antigen receptor	2YWY	UM6SQD	113	50
4	Lipase	1LGY	YTP3YC	269	213

##### Evaluating if mutants present a higher number of contacts

We hypothesized that if substituting a not interacting amino acid pair by another one interacting that was obtained from a known structure, the total number of inter-residues contacts would increase. Consequently, protein stability probably would increase. Hence, to evaluate if this hypothesis is correct, we proposed a test for wild and mutant structures using an independent tool for contacts measurement. We selected for this step the Arpeggio script tool [18].

For wild structures and their respective mutants, we calculated seven types of residue interactions: hydrogen bonds (HB), weak hydrogen bonds (Weak HB), ionic, aromatic, polar, weak polar, and hydrophobic interactions. We determined the contact number variation between each mutant and their wild structure. If the variation was positive, the number of contacts increased. If it was negative, the number of contacts reduced. Then, we determined the overall accuracy considering if the mutant structure presents a higher number of contacts or at least kept the number of contacts (Table 2; Tables S7, S8, S9, S10).

**Table 2:** The overall accuracy of mutants that present a higher number of contacts or at least keep the number of contacts when compared to the wild type (details were included in the supplementary material: Tables S7, S8, S9, S10)

Proteus ID	PDB ID	HB	Weak HB	Ionic	Aromatic	Polar	Weak polar	Hydrophobic^1^
UM6SQD	2YWY	0.84	0.66	0.96^2^	0.84^2^	0.94	0.98	0.28
N7Q9RZ	1LVB	0.97	0.79	1.0	0.9	0.95	1.0	0.26
YTP3YC	1LGY	0.77	0.66^2^	0.98^2^	0.9	0.96	1.0	0.31
4L8HLU	1BGA	0.79	0.42	0.84	0.77^2^	0.87	0.98	0.22

^1^For hydrophobic interactions, we also calculated the accuracy considering mutant structures that reduced or kept the number of contacts. For this case, UM6SQD presented an accuracy of 0.84; N7Q9RZ of 0.82; YTP3YC of 0.86; and 4L8HLU of 0.88. ^2^ Not statistically relevant (check supplementary material for details). HB: hydrogen bonds. The projects are available at http://proteus.dcc.ufmg.br/result/id/[**Proteus ID**]

Table 2 shows that most of the mutant structures presented an increment in the number of contacts (except for hydrophobic interactions, which was already expected). For example, Proteus found 50 mutation pairs for the immunoglobulin new antigen receptor (PDB ID: 2YWY; Proteus ID: UM6SQD). The contacts analysis indicates that 84% of these 50 structures (i.e. 42 mutants) presented an increment in the number of hydrogen bonds. Also, 66% of the mutants presented an increment in the total number of weak hydrogen bonds (when less restrictive parameters are considered for contacts determination). When the interactions between acceptor and donor are calculated without considering the torsion angle (Arpeggio calls it “polar” interaction), the number of contacts improved or kept in 94% of the structures (98% for weak polar interactions, i.e. when considered less restrictive parameters). Although ionic and aromatic interactions presented 96 and 84%, respectively, a hypothesis test (paired T-Test; single-tailed) indicated that the results were not statistically relevant.

ProteusDB was constructed using polar amino acids able to perform hydrogen bonds in the side-chain. Thus, an increment in the number of hydrophobic interactions based on Proteus predictions probably should not be related to a consequent improvement in protein stability. Hence, it was expected a reduction in the number of hydrophobic interactions, which agree with the low values presented in Table 2. On the other hand, we also calculated the accuracy considering the reduction or kept of hydrophobic interactions contacts as the expected value. Thus, we obtained an accuracy of 84% (UM6SQD), 82% (N7Q9RZ), 86% (YTP3YC), and 88% (4L8HLU).

#### Case study 2: mutations validated experimentally

In the second case study, we aimed to evaluated if any mutation proposed by Proteus had experimental evidence. Thus, we selected a list of single and double mutations experimentally validated for the structure of bacteriophage T4 lysozyme (PDB ID: 2LZM) in the ProTherm database [19–21]. We collected a total of 1716 mutations, their labels, ΔΔG experimentally estimated, temperature (T), pH, and the reference paper. We filtered the collected data to remove redundancy, lines with blank fields, and mutations with estimated ΔΔG higher than zero (346 mutations remained). Then, we ran Proteus for the lysozyme chain A structure and obtained a total of 272 mutation pairs suggested (Table S6). A total of 48 mutations were predicted with a negative value of ΔΔG by MAESTRO tool (negative values corresponding to stabilizing mutations).

Thereafter, we compared double mutations experimentally validated with the predicted results. However, most of the experimental double mutation data were prevenient from alanine scanning assays. Proteus does not consider mutations for alanine, due to this amino acid was not able to perform hydrogen bonds in the side-chain. Hence, it was not possible to provide a complete benchmarking of Proteus using ProTherm database. And, consequently, we did not find predicted mutation pairs obtained by Proteus with available data obtained experimentally. Thus, we decided to compare our predictions to single mutations reported in the ProTherm list (Table S11), for this presenting a wider range of amino acid substitutions when compared to the double mutations list (not only for alanine). After this comparison, we detected four predicted mutations with experimental evidence (Table 3).

**Table 3:** List of predicted mutations for the bacteriophage T4 lysozyme (PDB ID: 2LZM)

Predicted					Experimental		
Mutations suggested	**Template**			**ΔΔG**_**p**_	**Mutation validated**	**ΔΔG**_**e**_^a^	**Source**
	**PDB/chain**	**R1**	**R2**				
S44K/K48N	3lk4-W	K237	N241	− 0.432	S44K	− 0.2	[25]
K147E/T151Y	3ub9-A	E48	Y52	−0.415	K147E	−0.1	[26]
N40R/S44W	1w35-A	R163	W167	− 0.41	S44W	− 0.05	[25]
S44Q/K48Y	1c27-A	Q130	Y134	−0.201	S44Q	−0.27	[25]

Mutations predicted by Proteus (left). Mutations with experimental evidence (right). R1: first amino acid residue from the template where the mutation pair was extracted; R2: second amino acid residue from the template where the mutation pair was extracted; ΔΔG: Gibbs free energy variation (ΔΔGp: predicted; ΔΔGe: experimental). The predicted mutations are available at http://proteus.dcc.ufmg.br/result/id/SYLX52 (details were included in the supplementary material: Tables S6 and S11). ^a^ ΔΔG_e_ was multiplied for − 1 because of ProTherm uses a different ΔΔG definition when compared to MAESTRO. ProTherm considers positive values of ΔΔG as stabilizing, while MAESTRO considers negative values of ΔΔG as stabilizing. While one of them uses ΔG = G_folded_-G_unfolded_, the other uses ΔG = G_unfolded_-G_folded_

## Discussion

Proteus intended to become the first step in the mutation prediction process for proteins that do not have previous mutation studies, and which aim to introduce new intra-chain contacts without alteration of protein conformation. Our tool evaluates residues in a wild protein and suggests, based on the alignments between the pair residues and contacts in query databases, possible mutations in the residue pair in order to introduce a new (intra-chain) interaction between the proposed residues without altering the main conformation of the polypeptide chain.

Proteus suggests mutations in residue pairs if the main chain of these amino acids and their posterior and anterior neighbors are overlapped (cutoff below the set value of 0.5 Å), ensuring the conformation. The proposed mutations are based on other high-resolution three-dimensional structures, experimentally resolved and available in the PDB. Hence, if a given conformation of two amino acid residues and their target protein neighbors is found in the database, so it is suggested that these residues may be replaced.

### Comparison with other tools

Proteus presents a new strategy to suggest mutations using known structures from PDB. For this reason, we compared the functionalities of Proteus and other mutation tools (Table S1). Proteus’ primary goal is to suggest mutations based on known structures. For this, Proteus searches for target sites in the protein structure for possible mutations, distancing Proteus from sequence-based methods, such as MODIP and Mustab. Proteus is also different from other structure-based approaches such as SDM, mCSM, DUET, MAESTRO webtool, or other strategies used to propose mutations based on structural signatures (also called fingerprints) like SSV (structural signature variation) [15]. Hence, the main objective of this tools is to predict the free energy difference between mutant and wild type molecules. We did not find a tool with all characteristics of Proteus.

However, the Proteus software suite uses some of these tools, such as MAESTRO and SSV (used to provide faster searches) to best characterize the proposed mutations. The existence of other tools to perform the same task, but using different strategies, does not restrict their use together to help achieve accurate results. In conclusion, we suggest that Proteus can be used together with other tools to suggest more accurate mutations for experimental validation aiming to reduce costs.

### Case studies

In the first case study, we used Arpeggio, a tool with a different methodology for interactions determination, to evaluate changes in the number of contacts when wild and mutant proteins are compared. In the four examples evaluated, we could observe an increment in the number of hydrogen bonds and a reduction in the number of hydrophobic interactions in most mutants.

In the second case study, we tried to evaluate if some mutation proposed already present experimental validation in the literature. We found four mutations in which our predictions agree with experimental data. The effects of 19 different amino acid substitutions on the structure and stability of an alpha-helix of the S44 of the phage T4 lysozyme was previously established [25]. S44 is residue solvent-exposed and relatively free of interactions with neighboring residues, which may indicate that this residue is a potential target for mutation suggestions. Experimental data indicate that any amino acid substitution (with the exception of proline), causes little if any perturbation of the alpha-helix backbone [25]. This could indicate that the three substitutions indicated by Proteus (S44K, S44W, and S44Q) would not destabilize the protein structure (Table 3). In addition, the ΔΔG predicted values by MAESTRO agree with experimental predicted values, indicating a tendency to improve the stabilization. Furthermore, we highlighted that the predicted ΔΔG could not be directly compared to the experimental ΔΔG, since they correspond to ΔΔG for the mutation pair and ΔΔG for a single mutation, respectively. However, this could be the first evidence that the change of the side-chain does not cause disturbances that disfavor the protein structure stability.

We intend in the future to perform in vitro experiments to validate Proteus suggestions, especially for the improvement of enzymes used in the second-generation biofuel production.

## Implementation

### ProteusDB

We used Biopython [17, 27] and in-house scripts to detect the contacts in all three-dimensional structures collected. The list of atoms that could perform interactions, and their respective distance cutoffs, were established by Bickerton et al., (2011) when classifying hydrogen bonds, ionic interactions, and disulfide bonds (Table 4). In the present form, we only collected interactions between atoms from amino acid residues side chains.

**Table 4:** List of atoms that could perform interactions and distance cutoffs. Adapted from [28]

**Interaction**	**Distance**	**Atoms**
Hydrogen bond	< 3.50 Å	• **ARG**: NE, NH1, NH2
		• **ASN**: ND2, OD1
		• **ASP**: OD1, OD2
		• **GLN**: NE2, OE1
		• **LYS**: NZ
		• **GLU**: OE1, OE2
		• **HIS**: ND1, NE2
		• **SER**: OG
		• **THR**: OG1
		• **TRP**: NE1
		• **TYR**: OH
Ionic	< 6.00 Å	• **ARG**: CZ
		• **ASP**: CG, OD1, OD2
		• **GLU**: CD
		• **HIS**: CD2, CE1, CG
Disulfide bond	< 2.08 Å	• **CYS**: S

For every two amino acid residues involved in the interaction, we collected the coordinates of all their atoms and, in addition, the coordinates of main-chain atoms of their anterior and posterior residues, generating the triad pair. We saved each triad pairs (a file in PDB format) in folders named according to the amino acid pair involved in the interaction (for example, an interaction D100-K200 would be saved in the folder ASP, while an interaction K300-D400 would be saved in the folder LYS). Among all twenty amino acids, twelve of them were considered in this work due to their potential to perform specific interactions as previously described (Table 1): arginine (ARG/R), asparagine (ASN/N), aspartate (ASP/D), glutamine (GLN/Q), lysine (LYS/K), glutamate (GLU/E), histidine (HIS/H), serine (SER/S), threonine (THR/T), tryptophan (TRP/W), tyrosine (TYR/Y), and cysteine (CYS/C). Therefore, we constructed 122 folders (the combination of the first 11 amino acids, plus one folder to contain disulfide bonds).

**Fig. 4:**
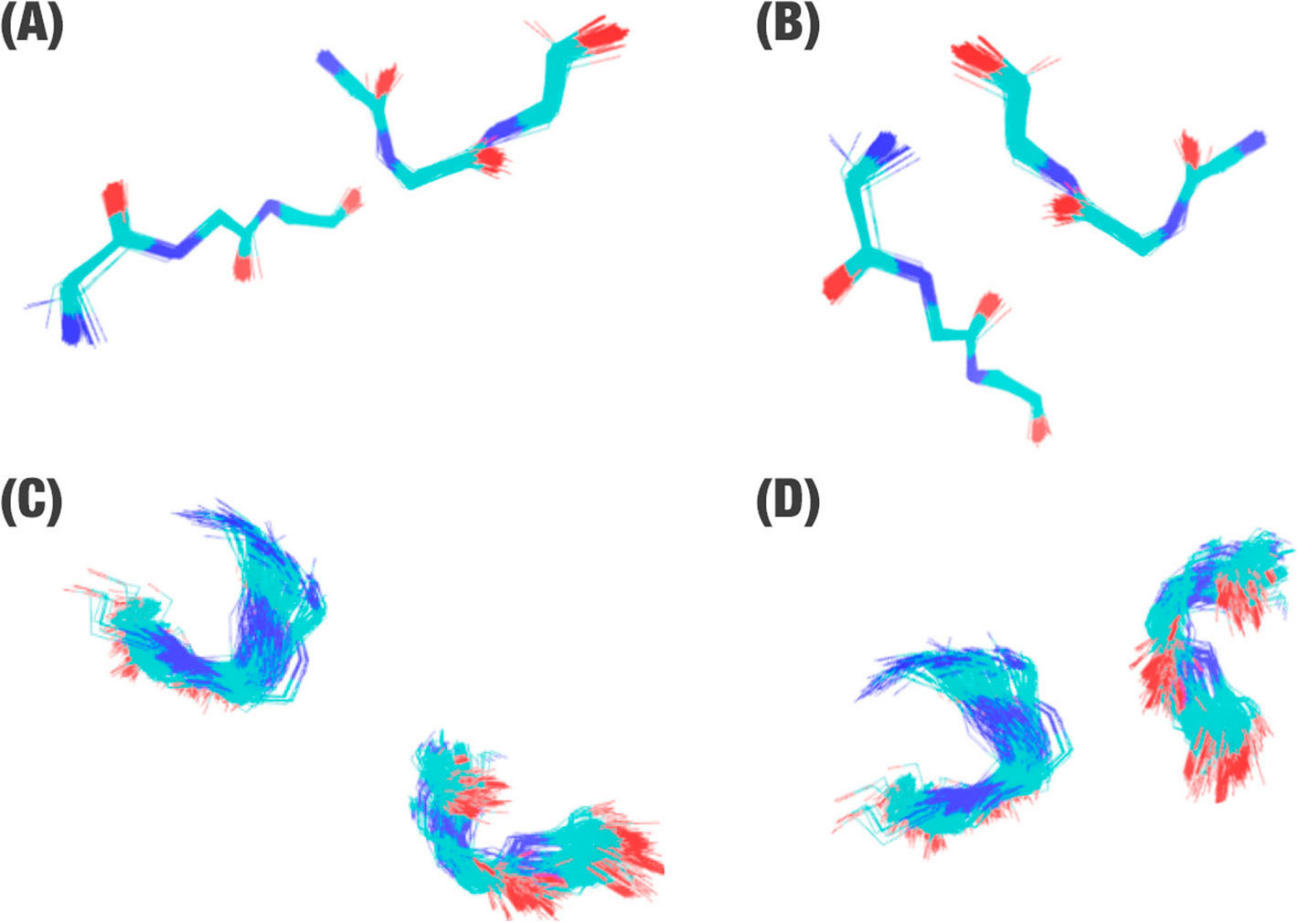
Example of cysteine cluster. In this example, we can see two CYS-CYS clusters (**a**-**b**) and two ASP-ARG clusters (**c**-**d**), which were obtained from the structural alignment of all triad pairs with an RMSD score of 0.5 Å

To reduce redundancy of triad pairs in ProteusDB, we clustered them. For this step, we used the gmx cluster 9 tool from GROMACS 10 software [29, 30]. We used a single linkage algorithm at gmx tool for clustering structures based on the RMSD (Root Mean Square deviation) cutoff ranging from 0.3–0.9 Å (values defined empirically; Fig. 4).

After the clustering step, the first element in each cluster was defined as its representant. Clusters were regrouped in 12 databases based on the first amino acid in contact (N) and stored in a MySQL database management system aim to improve the search performance. The 12 databases, which together were called the ProteusDB, have a total of 175,267 representants of triad pair structures, being CYS (database of cysteine contacts) the smallest database with 2552 structures, and ASP (a database with aspartate contacts) the biggest with 35,980 structures (Table 5).

**Table 5:** The number of representants of triad pair structures in each database that forms ProteusDB

Database name	Amino acid	Number of structures
ARG	Arginine	28,785
ASN	Asparagine	12,123
ASP	Aspartate	35,980
CYS	Cysteine	2552
GLN	Glutamine	8800
GLU	Glutamate	24,828
HIS	Histitine	15,440
LYS	Lysine	11,348
SER	Serine	13,494
THR	Threonine	9947
TRP	Tryptophan	2458
TYR	Tyrosine	9512
**Total**		**175,267**

It is important to highlight that the CYS database contains only contacts between CYS-CYS amino acid residues, while all other databases contain contacts among one specific residue and all the other amino acids. For example, the ARG database contains all the following contacts: ARG-ARG, ARG-ASN, ARG-ASP, ARG-GLN, ARG-GLU, ARG-HIS, ARG-LYS, ARG-SER, ARG-THR, ARG-TRP, and ARG-TYR. In the future, we intend to include contact ARG-CYS.

### ProteusWEB

We constructed a user-friendly interface, herein called ProteusWEB, to provide a secure method to run the Proteus algorithm. ProteusWEB was constructed using the PHP, HTML, and JavaScript languages, using a similar architecture to [15, 31–33]. 3Dmol.js was used for visualization of protein structures [16].

### PSE (Proteus search engine)

#### Defining target triad pairs

**Fig. 5:**
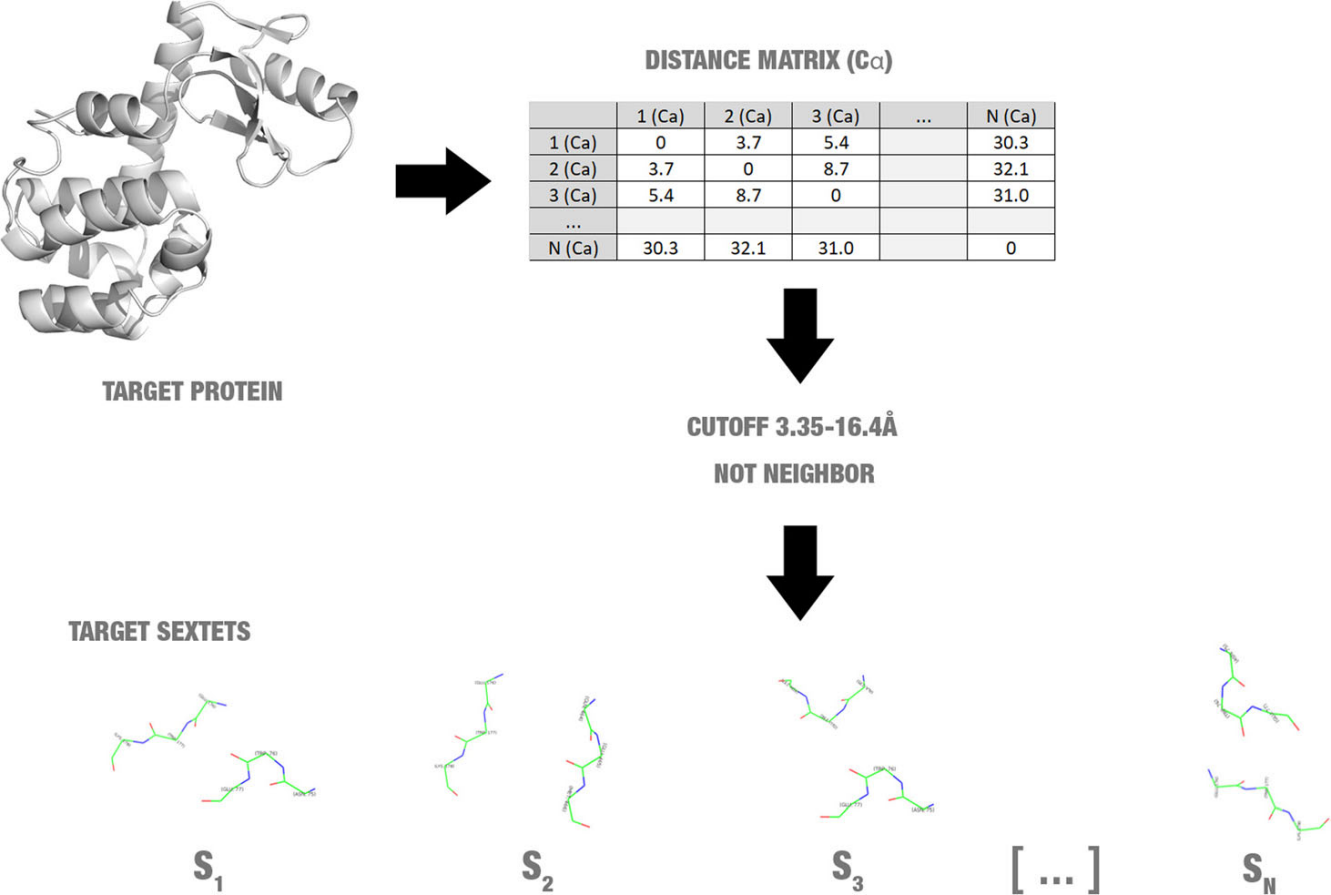
Detection of target triad pairs (also called target sextets)

To define target triad pairs for starting the search, Proteus analysis the distance between all alpha carbon atoms of the protein. Proteus defines amino acid pairs, which are not neighbours and have alpha carbons at a distance between 3.35 and 16.4 Å are a target for mutations (Fig. 5). This distance range was defined based on the minimum and maximum distance value of alpha carbons of residues interacting found in ProteusDB.

#### Search

**Fig. 6:**
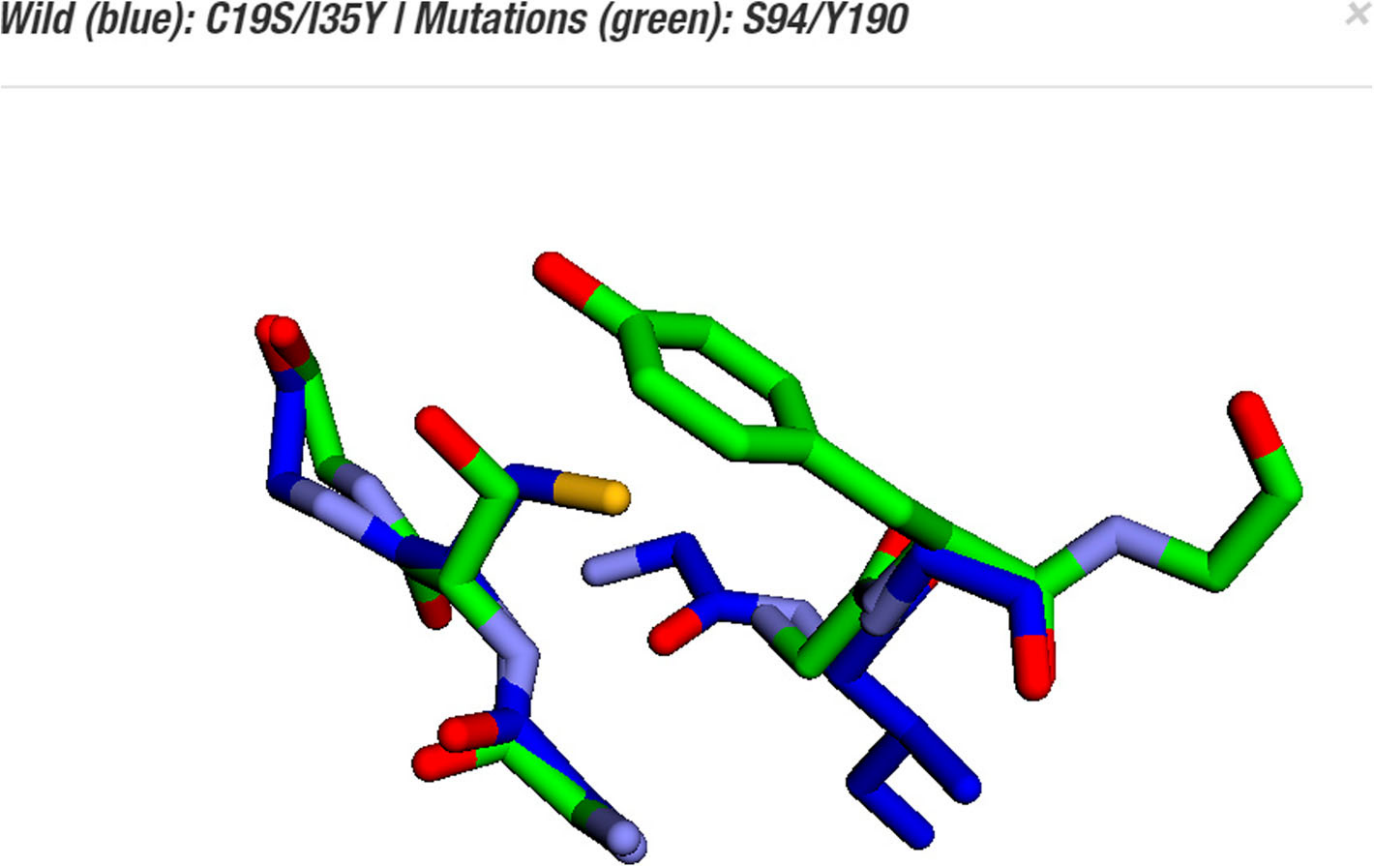
Example of structural alignment between triad pairs. In this case, Proteus suggested mutations for the sites C19-I35 (blue sticks) of the crystal structure of tobacco etch virus protease (PDB ID: 1q31). Proteus detected overlap with RMSD of 0.46 between the C19-I35 residues and triad pairs formed between S94-Y190 (green sticks) of a membrane protein (PDB ID: 1iiw). For this reason, Proteus suggested the mutation C19S-I35Y

To determine if a mutation could be suggested, Proteus performs structural alignment between triad pairs of a target protein and the triad pairs of ProteusDB. Structural alignment allows the comparisons between the shape of macromolecules. We hypothesized that if the main-chain conformation of six residues is conserved, a double mutation change is possible (Fig. 6).

The differences between structures are evaluated using RMSD (Root Mean Square deviation). RMSD is the average value for the average deviation of the atoms from a structure when compared to another (eq. 1). The lower this value, the most similar are both structures.

1 $$ RMSD=\sqrt{\frac{1}{N}\sum \limits_{i=1}^N{\delta}_i^2} $$

Where *δ*_i_ is the difference between coordinates of an atom *i* and the equivalent in another structure for N atoms. Proteus algorithm compares the 24 atoms of each triad pairs (C, N, O, and Cα from each main chain of six amino acids).

However, for each target triad pairs, Proteus had to perform 175,267 comparisons between structures (total of ProteusDB’s structures), which requires high computational costs. The structural alignment is a vital step for Proteus to suggest mutations. Therefore, this step cannot be removed, but some strategies with lower computational costs could be used to reduce the number of structural alignments removing for this comparison, triad pairs with low possibilities to present similar structures. For this step, we used structural signatures (also called fingerprints).

#### Using structural signatures to reduce the structural comparisons

After preliminary tests, we observed that the structural alignment between a target triad pair and all structures from ProteusDB presented a high computational cost. For instance, the structure of bacteriophage T4 lysozyme (PDB ID: 2LZM) presents a sequence with 164 amino acids and approximately 1000 amino acid pairs target for mutation suggestions based on a preliminary Proteus analysis. The structural alignment between 2LZM’s target triad pairs and all structures of ProteusDB taken almost 1 week using 32 CPUs (data not shown). However, we noticed that some comparisons performed by structural alignment were unnecessary. For instance, the distance between the main chain atoms for the same amino acids is almost conserved (distances between C, N, O, and Cα), but the structural alignment promotes these comparisons.

**Fig. 7:**
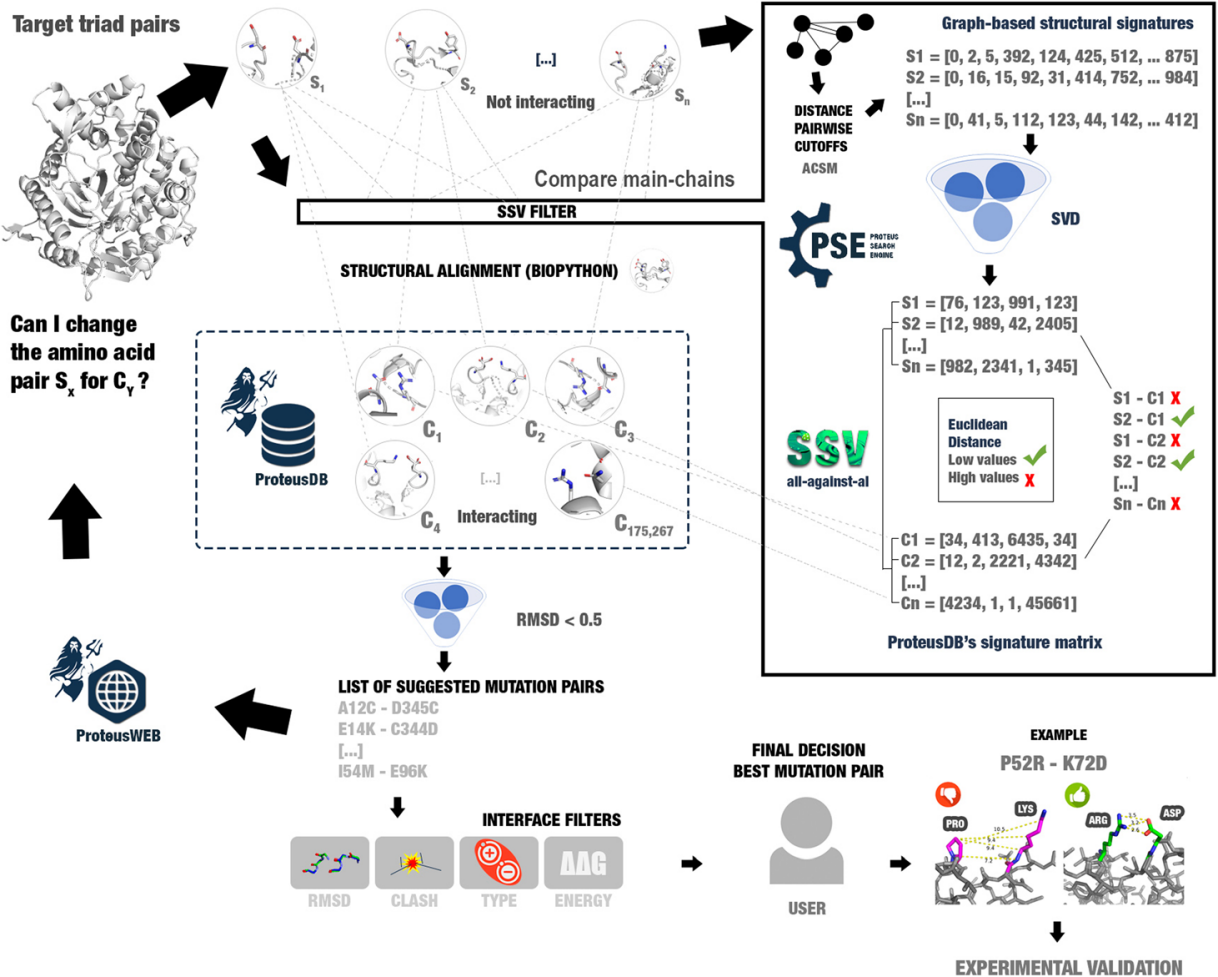
Proteus complete pipeline. Structural alignment has a high computational cost. Hence, we used a filter step to reduce the number of structural alignments. The filter step uses the variation of structural signatures (fingerprints) to remove structures from ProteusDB with a low possibility to be like target triad pairs. SSV (structural signature variation) uses Euclidean distance between four-dimensional vectors, which has lower computational cost than structural alignment. This accelerates the search for similar structures considerably

Hence, to reduce the computational costs, we introduced a filter step before the structural alignment using the SSV (Structural Signature Variation) method [15]. SSV is a graph-based methodology for three-dimensional structure comparisons using structural signatures, linear algebra techniques, and the variation of vector distances. The SSV algorithm suggests that molecules with similar structures have similar structural signatures. Therefore, the Euclidean distance between signatures of different macromolecules could be used to define if a macromolecule is more like a model macromolecule than another one [15]. Hence, we introduced the SSV filter before the structural alignments step (Fig. 7).

SSV uses aCSM (atomic Cutoff Scanning Matrix) to construct structural signatures [34]. In aCSM method, a protein structure is converted in a graph, where the atoms are the vertices and edges are defined by a set of distance cutoffs between atoms. The pairwise atoms are calculated, also analyzing the pharmacophore atom properties. Lastly, a signature vector (fingerprint) that represents the protein structure is constructed.

To construct the ProteusDB triad pairs signature, we used aCSM with parameters cutoff minimum distance of 0 Å, a maximum cutoff distance of 10 Å, and a cutoff distance step of 0.1 Å – parameters defined in [15]. This generated a matrix of 175,267 lines (structures) and 576 columns (features).

We constructed signature vectors for each triad pair of ProteusDB. Besides, to reduce noise and boost the search process, we performed dimensionality reduction using SVD (Singular Value Decomposition). SVD is a technique from linear algebra for noise-reducing based on analysis of multivariate data and rearrangement of the vector space for retrieve non-evident relationships between the matrix elements [35]. In SVD, a real or complex matrix A (of length m x n) is factored in three other matrixes, where the matrix U is an m x m unitary matrix, S is an m x n rectangular diagonal matrix with the singular values, and V is a n x n transposed unitary matrix (eq. 2).

2 $$ A= US{V}^T $$

The plot of matrix S was used to analyze the number of singular values. We calculated that four dimensions were necessary to represent the ProteusDB’s signature matrix (70% of the singular values presented in matrix S), i.e. the 576 representative values for each triad pair were reduced for only four. Also, we used SVD matrixes to calculate the reduced vectors for each new target triad pairs (see methodology details in Silvério-Machado et al., 2015; Pires et al., 2013).

To calculate similarity, SSV used Euclidean distance between target triad pairs reduced signatures and all reduced signatures of ProteusDB. Despite SSV presented a fast comparison method with a high number of true positives, we observed a high number of false positives (data not shown). Therefore, we used SSV only as a filter to reduce the number of structural comparisons. For example, in the case study with 2LZM protein, the SSV filter reduced the number of probable structures with considerable signature differences. For each triad pairs defined as a possible target for mutations, Proteus did not need to perform a structural alignment with each element of ProteusDB. Using a simple Euclidean distance between the reduced signature vector, we were able to define if a structure has a chance to be like another. We defined that a maximum distance of cutoff was 13 (maximum distance used to include all possible mutations suggested for 2LZM). A Euclidean distance between a reduced signature and all ProteusDB could be executed in less than one second and reduced the number of structural alignments from 700 million to 2 million. Thus, we obtained the same result in less than one day using only one CPU (data not shown).

Although the search for mutations using only structural alignment and using SSV followed of a reduced search for similar main chain structures have presented the same result in our tests, with considerable difference of running time, we cannot affirm that the SSV filter could remove a true positive result for the structural alignment step in another protein. However, we believe that the SSV filter turns viable the large-scale use of Proteus as a required for a web server application.

#### ΔΔG and clash

Proteus shows a Gibbs free energy estimation calculated by the MAESTRO command-line tool [6]. MAESTRO receives as input a PDB and a pair of mutations. MAESTRO returns a ΔΔG predicted value, which represents the total predicted change of stability (kcal/mol). ΔΔG values lower than zero indicates stabilizing mutations and ΔΔG values higher than zero destabilizing.

Furthermore, stereochemistry clash is detected when the performed changing of the side chain atoms for the suggested mutations, inserts another atom a non-allowed position. To detect this, we calculated the Euclidean distance between the coordinates of the new atoms and the neighborhood. We defined that when an inserted atom crosses a cutoff distance of 2 Å of any one atom from the neighborhood, a possible stereochemistry clash is reported.

Despite the main objective of Proteus is to suggest stabilizing mutations and without clash, we expect most of the predicted mutations would present destabilizing changes or stereochemistry clash. Mutations that insert stereochemistry clashes could be better evaluated using high computational cost strategies, like molecular dynamics. Hence, we decided to hide clashes.

### Case studies

#### Data collection

We collected the structures of an immunoglobulin new antigen receptor (PDB ID: 2YWY), a protease (PDB ID: 1LVB), a lipase (PDB ID: 1LGY), a β-glucosidase (hydrolase; PDB ID: 1BGA), and a lysozyme (PDB ID: 2LZM) from Protein Data Bank (PDB) [36]. Protein structures were parsed using the PDB module from Biopython [17, 27]. We removed heteroatoms and used only chain A in the following steps.

#### Mutations prediction

We submitted the five structures for ProteusWEB interface. The results were visualized using the Google Chrome browser (version 79.0.3945.130 64 bits) and were analyzed using in-house scripts developed with the Python programming language. The results can be accessed in the ProteusWEB interface by the link <http://proteus.dcc.ufmg.br/result/id/[Proteus_ID]>, where the Proteus ID of the immunoglobulin new antigen receptor (2YWY) is UM6SQD, the Proteus ID of the protease (1LVB) is N7Q9RZ, the Proteus ID of the lipase (1LGY) is YTP3YC, the Proteus ID of the β-glucosidase (1BGA) is 4L8HLU, and the Proteus ID of the lysozyme (2LZM) is SYLX52.

#### Determining contacts using arpeggio software

We executed the Arpeggio software [18] to determine the total of inter-residue contacts in wild and mutants’ structures used in the first case study. We considered seven types of contacts in the posterior analysis: hydrogen bonds, weak hydrogen bonds, ionic, aromatic, polar, weak polar, and hydrophobic interactions.

For hydrogen bonds, Arpeggio considers residues with acceptor and donor atoms in a cutoff distance up to 3.9 Å, the polar distance up to 3.5 Å, an angle radian of 1.57, and angle degree of 90°. The weak hydrogen bond interaction (Weak HB) is determined by less restrictive values: angle radian of 2.27 (allowing variations of 0.52–2.62), angle degree of 130° (allowing variations of 30–150), but a lower distance value (3.6 Å). Polar and weak polar contacts use hydrogen-bonding definitions, but not considering the angle criteria.

Arpeggio considers ionic interactions as strong electrostatic bonding between atoms of two ions of opposing charges in a distance cutoff up to 4.0 Å. Aromatic interactions occur among aromatic ring structures with other rings, atoms, or groups. Jubb et al. [18] reported that electron-rich ring faces could present a partial negative and electron-deficient ring edges could present a partial positive charge. This could be compared to hydrogen bond acceptors. Arpeggio considers aromatic atoms in a distance up to 4.5 Å and a centroid distance between rings up to 6.0 Å. Finally, hydrophobic interactions refer to an aggregation of hydrophobic moieties. Arpeggio considers as hydrophobic interactions, apolar atoms at a distance up to 4.5 Å.

We determined the total number of contacts parsing the *.sift files using in-house scripts. Then, we calculated the variation of the total of each type of contact. To determine if the variation was statistically relevant, the *p*-value was calculated using T-Test function (parameters: paired and single-tailed) from Microsoft Excel software (16.0.12430.20112 32 bits).

#### ProTherm

We analyzed the ProTherm database [19–21] searching for stabilizing mutations to verify if some suggested mutation by Proteus had experimental data available. We selected the structure of bacteriophage T4 lysozyme (PDB ID: 2LZM) to work in this case study because this structure presented the highest number of mutations reported in ProTherm (1716 results). We filtered this list to remove redundancy, lines with blank fields, and mutations with estimated ΔΔG higher than zero (439 mutations remain: 346 single, 50 double, and 43 three or more mutation sites). From the 50 double mutations, 26 were mutations for amino acids not available in ProteusDB (being 17 substitutions for alanine). Then, we compared the double mutations from ProTherm with the predicted results by Proteus, but we did not find a perfect double match. Thereby, we decided to use the 346 single mutants from ProTherm (see supplementary material). We found four results with ΔΔG lower than zero that match with the predicted data. MAESTRO and ProTherm use a different definition of ΔΔG. While one of them uses ΔG = G_folded_-G_unfolded_, the other uses ΔG = G_unfolded_-G_folded_. ProTherm considers positive values of ΔΔG as stabilizing, while MAESTRO considers negative values ones as stabilizing. Therefore, we multiplied the ProTherm values by − 1 to compare the results.

## Conclusion

Here, we presented Proteus, a new computational method for proposing mutation pairs in a target 3D structure based on introducing new side-chain interactions in the macromolecule. We believe that Proteus’ algorithm could suggest new mutations that conserve or improve the stability of the target proteins. Combined with computational approaches, such as sequence conservation, molecular dynamics, and mutation impact prediction, Proteus could give new insights into the rational design of engineered proteins.

### Availability and requirements

**Project name:** Proteus.

**Project home page:**http://proteus.dcc.ufmg.br

**Operating system(s):** Platform independent (web-based tool).

**Programming language:** Python, PHP, JavaScript.

**Other requirements:** Google Chrome browser.

**License:** CC-BY.

**Any restrictions to use by non-academics:** None.

## Supplementary information

**Additional file 1: **Supplementary **Tables S1-S11.Table S1.** Comparison of mutation tools. **Table S2.** Fifty mutants suggested for the immunoglobulin (2YWY). **Table S3.** Sixty-one mutants suggested for the Protease (1LVB). **Table S4.** Two-hundred and thirteen mutants suggested for the Protease (1LGY). **Table S5.** Three-hundred forty-four mutants suggested for the β-glucosidase (hydrolase; 1BGA). **Table S6.** Two-hundred seventy-two mutants suggested for the lysozyme (2LZM) used in the second case study. **Table S7.** The total number of contacts for the wild protein and their 50 mutants of the immunoglobulin (2YWY). **Table S8.** The total number of contacts for the wild protein and their 61 mutants of the Protease (1LVB). **Table S9.** The total number of contacts for the wild protein and their 213 mutants of the Protease (1LGY). **Table S10.** The total number of contacts for the wild protein and their 344 mutants of the β-glucosidase (hydrolase; 1BGA). **Table S11.** List of stabilizing mutations for the PDB: 2LZM collected from ProTherm.

## Data Availability

All data generated or analysed during this study are included in this published article: additional file 1 (supplementary Tables S1-S11).

## Abbreviations

aCSM: Atomic cutoff scanning matrix
HB: Hydrogen bonds
PDB: Protein data bank
Proteus: Protein engineering supporter
ProteusDB: Proteus data bank
PSE: Proteus search engine
RMSD: Root mean square deviation
SSV: Structural signature variation
SVD: Singular value decomposition
T: Temperature
