# Supplementary material for: Proteus: An algorithm for proposing stabilizing mutation pairs based on interactions observed in known protein 3D structures
Source: BMC Bioinformatics. 2020 Jul 1;21:275. doi: 10.1186/s12859-020-03575-6 (PMC7330979; doi:10.1186/s12859-020-03575-6)
Supplement: Supplementary file 1 — Additional file 1: Supplementary Tables S1-S11.Table S1. Comparison of mutation tools. Table S2. Fifty mutants suggested for the immunoglobulin (2YWY). Table S3. Sixty-one mutants suggested for the Protease (1LVB). Table S4. Two-hundred and thirteen mutants suggested for the Protease (1LGY). Table S5. Three-hundred forty-four mutants suggested for the β-glucosidase (hydrolase; 1BGA). Table S6. Two-hundred seventy-two mutants suggested for the lysozyme (2LZM) used in the second case study. Table S7. The total number of contacts for the wild protein and their 50 mutants of the immunoglobulin (2YWY). Table S8. The total number of contacts for the wild protein and their 61 mutants of the Protease (1LVB). Table S9. The total number of contacts for the wild protein and their 213 mutants of the Protease (1LGY). Table S10. The total number of contacts for the wild protein and their 344 mutants of the β-glucosidase (hydrolase; 1BGA). Table S11. List of stabilizing mutations for the PDB: 2LZM collected from ProTherm. [file 12859_2020_3575_MOESM1_ESM.pdf]

Supplementary material

"*Proteus: An algorithm for proposing stabilizing mutation pairs based on interactions observed in known protein 3D structures*"

Tables

Table S1. Comparison of mutation tools. 2
Table S2. Fifty mutants suggested for the immunoglobulin (2YWY). 3
Table S3. Sixty-one mutants suggested for the Protease (1LVB). 6
Table S4. Two-hundred and thirteen mutants suggested for the Protease (1LGY). 9
Table S5. Three-hundred forty-four mutants suggested for the β-glucosidase (hydrolase; 1BGA).... 20
Table S6. Two-hundred seventy-two mutants suggested for the lysozyme (2LZM) used in the second case study. 36
Table S7. The total number of contacts for the wild protein and their 50 mutants of the immunoglobulin (2YWY). 49
Table S8. The total number of contacts for the wild protein and their 61 mutants of the Protease (1LVB). 51
Table S9. The total number of contacts for the wild protein and their 213 mutants of the Protease (1LGY). 53
Table S10. The total number of contacts for the wild protein and their 344 mutants of the β-glucosidase (hydrolase; 1BGA). 59
Table S11. List of stabilizing mutations for the PDB: 2LZM collected from ProTherm. 68

Table S1. Comparison of mutation tools.

TOOL	INPUT	DESCRIPTION	REFERENCE
**PROTEUS**	Structures	Suggests mutation pairs based on known structures found in PDB.	-
**SSBOUND**	Structures	Inserts new disulfide bonds, which could improve the thermostability.	(Hazes and Dijkstra, 1988)
**MODIP**	Structures	It uses a classification system based on conformation parameters for disulfide bonds.	(Sowdhamini *et al.*, 1989)
**MUSTAB**	Sequence	Uses machine learning to predict protein stability changes upon amino acid substitutions.	(Teng *et al.*, 2010)
**IPTREE-STAB**	Sequence	Predicts protein stability changes ($\Delta\Delta G$) upon single amino acid substitutions.	(Huang *et al.*, 2007)
**POPMUSIC**	Structures	Predicts thermodynamic stability changes using a linear combination of statistical potentials based on the solvent accessibility of the mutated residue.	(Dehouck *et al.*, 2011)
**SDM**	Structures	uses substitution probability tables obtained from known 3-D structures to analyze the variation of amino acid replacements tolerated within the family of homologous proteins	(Pandurangan *et al.*, 2017)
**DUET**	Structures	Combines the methods SDM and mCSM in a hybrid approach to try to obtain a more accurate prediction of $\Delta\Delta G$.	(Pires *et al.*, 2014a)
**MCSM**	Structures	mCSM uses graph-based signatures and pharmacophore proprieties in a machine learning approach to predict stabilizing and destabilizing mutations.	(Pires *et al.*, 2014b)
**SSV**	Structures	SSV uses graph-based structural signatures and compares Euclidian distances between vectors to predict if mutations insert similar characteristics to reference proteins.	(Mariano *et al.*, 2019)
**MAESTRO**	Structures	Combines high throughput scanning for multi-point mutations, prediction of free energy change ($\Delta\Delta G$) values, and stabilizing disulfide bonds.	(Laimer *et al.*, 2015)

Table S2. Fifty mutants suggested for the immunoglobulin (2YWY).

Proteus ID: UM6SQD. Available at <http://proteus.dcc.ufmg.br/result/id/UM6SQD>.

Mutation	Template	Chain	R1	R2	RMSD	ΔΔG	Clash
E57H/F66T	4r7k	C	H115	T131	0.46	2.601	No
E57N/F66S	1c27	A	N95	S110	0.35	4.303	No
E57Q/F66T	1h1i	B	Q83	T95	0.39	2.649	No
E57S/L68T	3oh8	A	S6	T106	0.26	3.624	No
F87Q/E101S	4wm8	B	Q111	S231	0.47	3.852	No
G15S/R74Q	3v6o	D	S17	Q88	0.38	0.746	No
I20T/I70K	1naq	A	T41	K67	0.45	4.326	No
K12E/L108Q	1uqt	B	E261	Q294	0.45	3.595	No
L18S/L73S	2ial	C	S18	S76	0.42	4.617	No
L18W/I70Q	4h3l	A	W93	Q109	0.44	3.597	No
L31E/A85D	5fgp	A	E43	D59	0.4	3.537	No
L31S/A85D	3sc7	X	S366	D509	0.48	4.284	No
Q5E/I20N	2ofc	B	E113	N121	0.5	4.086	No
Q5K/I20R	4cb6	B	K339	R355	0.47	2.694	No
Q5N/C22S	3o41	L	N161	S177	0.3	3.307	No
Q5S/C22T	3oh8	A	S6	T106	0.48	3.007	No
Q5T/I20H	3fe4	A	T214	H229	0.45	2.84	No
Q5T/I20N	3lw2	A	T93	N167	0.46	3.441	No
R38H/I49N	5f9o	H	H35	N99	0.49	2.196	No
T11H/T109R	4kpl	D	H124	R149	0.46	0.02	No
T11K/T109H	4jl1	A	K130	H176	0.49	0.581	No

Mutation	Template	Chain	R1	R2	RMSD	ΔΔG	Clash
T11N/K111T	3ecj	B	N157	T205	0.46	0.458	No
T11S/K111Q	4xk2	A	S85	Q126	0.28	0.144	No
T19E/R69N	3sys	A	E343	N369	0.32	1.184	No
T19E/S67N	2ofc	B	E113	N121	0.48	1.669	No
T19K/R69K	1get	B	K372	K392	0.48	1.921	No
T19N/R69S	3o41	L	N161	S177	0.29	1.524	No
T19S/R69T	3oh8	A	S6	T106	0.44	0.913	No
T34K/A85K	1get	B	K372	K392	0.29	2.168	No
T34N/A85S	3o41	L	N161	S177	0.32	2.77	No
T58S/S67Y	1juh	D	S105	Y215	0.37	0.261	No
T80S/V107T	3oh8	A	S6	T106	0.45	1.778	No
T80S/V107Y	1k8k	D	S166	Y222	0.4	1.863	No
T80W/V107D	2v8h	C	W251	D320	0.48	2.411	No
T80W/V107H	2qqz	A	W49	H58	0.49	1.411	No
V3N/C22S	1c27	A	N95	S110	0.44	4.414	No
V3S/L24T	3oh8	A	S6	T106	0.41	4.368	No
V56E/R69Q	1cpn	A	E51	Q61	0.46	1.862	No
V56N/R71K	3c46	A	N655	K665	0.47	1.416	No
V59E/F66N	3sys	A	E343	N369	0.42	3.536	No
V59N/F66S	3o41	L	N161	S177	0.48	3.682	No
W36D/Y81K	1gd1	R	D241	K306	0.34	4.48	No
W36H/C83K	1yi7	A	H133	K139	0.47	4.107	No
W36S/C83T	3oh8	A	S6	T106	0.47	4.376	No

Mutation	Template	Chain	R1	R2	RMSD	ΔΔG	Clash
W36T/Y81N	3lw2	A	T93	N167	0.34	4.515	No
Y37Q/E46Q	2f3q	A	Q168	Q175	0.49	3.175	No
Y37Q/K102E	2ic2	A	Q501	E561	0.33	2.885	No
Y55H/I70N	4qbu	A	H59	N87	0.41	4.164	No
Y55N/L68T	4dol	A	N163	T180	0.37	4.262	No
Y86S/G100T	3oh8	A	S6	T106	0.45	3.776	No

Showing 1 to 50 of 50 entries

Table S3. Sixty-one mutants suggested for the Protease (1LVB).

Proteus ID: N7Q9RZ. Available at <http://proteus.dcc.ufmg.br/result/id/N7Q9RZ>.

Mutation	Template	Chain	R1	R2	RMSD	ΔΔG	Clash
Q58W/V63H	2qqz	A	W49	H58	0.42	-0.303	No
T22S/S31Y	1k8k	D	S166	Y222	0.42	-0.251	No
L56W/V63H	3i24	B	W34	H93	0.45	-0.075	No
P13W/T17H	1i5z	A	W13	H17	0.46	-0.074	No
T22S/S31T	3oh8	A	S6	T106	0.48	0.004	No
Q58S/V63Y	1k8k	D	S166	Y222	0.5	0.052	No
T22K/S31R	2amu	A	K57	R73	0.42	0.074	No
L56Y/K65Q	4ch9	A	Y444	Q462	0.44	0.083	No
T22W/L56H	3aeh	B	W1180	H1204	0.43	0.102	No
T22Q/S31T	1lj5	A	Q322	T339	0.25	0.134	No
H20Q/S31T	1h1i	B	Q83	T95	0.37	0.188	No
T22Q/T29W	1qwy	A	Q244	W257	0.39	0.188	No
S208N/K215T	4dol	A	N163	T180	0.42	0.234	No
F132R/K141Q	3p9l	A	R234	Q242	0.46	0.385	No
L56S/K65Y	1k8k	D	S166	Y222	0.39	0.417	No
T22D/T29W	4m2f	A	D131	W318	0.46	0.426	No
Q58N/V63S	3o41	L	N161	S177	0.41	0.469	No
K141E/Y178W	2e2v	A	E452	W495	0.39	0.483	No
K99R/F162R	2hhc	A	R5	R34	0.49	0.535	No
E24N/T29S	3o41	L	N161	S177	0.5	0.546	No
L56W/K65H	2qqz	A	W49	H58	0.31	0.553	No

Mutation	Template	Chain	R1	R2	RMSD	ΔΔG	Clash
V209S/V216H	3nrq	B	S71	H115	0.48	0.584	No
T22E/S31N	3sys	A	E343	N369	0.38	0.635	No
T22D/T29K	1gd1	R	D241	K306	0.46	0.662	No
Q58K/V63R	2amu	A	K57	R73	0.38	0.673	No
Q58W/V63D	2v8h	C	W251	D320	0.35	0.71	No
N185Q/L189R	3px4	A	Q656	R660	0.44	0.832	No
T22N/T29S	1c27	A	N95	S110	0.44	0.855	No
Q58S/V63T	3oh8	A	S6	T106	0.48	0.885	No
G36S/I41T	3oh8	A	S6	T106	0.48	0.93	No
H20D/S31K	1gd1	R	D241	K306	0.44	0.939	No
L56S/K65T	3oh8	A	S6	T106	0.42	0.975	No
L56K/K65R	2amu	A	K57	R73	0.46	1.005	No
L56D/V63K	1gd1	R	D241	K306	0.38	1.02	No
T22K/Q58K	1h84	C	K241	K250	0.43	1.082	No
L56Q/V63T	1h1i	B	Q83	T95	0.47	1.298	No
N23K/L55R	2amu	A	K57	R73	0.44	1.426	No
N23S/L55T	3oh8	A	S6	T106	0.38	1.574	No
L56N/K65S	3o41	L	N161	S177	0.42	1.682	No
Q73R/W198Y	1es4	A	R30	Y151	0.49	1.749	No
L21Y/L55S	2zws	A	Y148	S183	0.49	1.802	No
K141Q/Y178N	2jjo	A	Q292	N313	0.47	1.95	No
P154K/I163T	2icc	A	K36	T47	0.49	2.151	No
P154S/I163H	2blv	A	S32	H40	0.41	2.204	No

Mutation	Template	Chain	R1	R2	RMSD	ΔΔG	Clash
I41S/I85W	2bi0	A	S311	W330	0.5	2.237	No
P154T/I163S	4wjk	A	T263	S272	0.43	2.461	No
Q74S/I85T	3oh8	A	S6	T106	0.39	2.649	No
L21H/L55K	5h8t	A	H116	K127	0.43	2.819	No
V112T/V156Q	4jf8	A	T177	Q192	0.48	2.882	No
Q74N/I85S	3o41	L	N161	S177	0.47	3.218	No
L111Q/L155H	3r0f	B	Q19	H24	0.48	3.314	No
L21T/L55N	3lw2	A	T93	N167	0.42	3.371	No
L21E/L55N	2ofc	B	E113	N121	0.43	3.438	No
I41E/I85Q	1cpn	A	E51	Q61	0.4	3.525	No
I42Q/I84N	2jjo	A	Q292	N313	0.43	3.53	No
L21S/V57T	3oh8	A	S6	T106	0.37	3.718	No
I35S/I42H	3nrq	B	S71	H115	0.44	3.722	No
L21N/L55S	1c27	A	N95	S110	0.5	3.872	No
L21N/V57S	3o41	L	N161	S177	0.19	3.894	No
I42S/I84T	3oh8	A	S6	T106	0.38	4.015	No
I42N/I84S	3o41	L	N161	S177	0.48	4.049	No

Showing 1 to 61 of 61 entries

Table S4. Two-hundred and thirteen mutants suggested for the Protease (1LGY).

Proteus ID: YTP3YC. Available at <http://proteus.dcc.ufmg.br/result/id/YTP3YC>.

Mutation	Template	Chain	R1	R2	RMSD	ΔΔG	Clash
G111R/S115W	1w35	A	R163	W167	0.36	-0.788	No
G111Y/S115T	4cae	C	Y88	T92	0.3	-0.605	No
K138D/S171Y	3pfc	A	D26	Y101	0.36	-0.527	No
G111Q/S115Y	1c27	A	Q130	Y134	0.23	-0.468	No
T11Q/T18R	4ni8	A	Q13	R20	0.31	-0.454	No
T11S/Q15D	4iqg	B	S37	D41	0.48	-0.388	No
V41T/K45S	3hh1	C	T43	S47	0.48	-0.38	No
Y187R/S191R	1qtx	B	R12	R16	0.35	-0.325	No
G111Q/S115R	2x1c	B	Q196	R200	0.24	-0.282	No
G111K/S115H	2ifq	A	K39	H43	0.28	-0.191	No
G111T/S115R	3mux	A	T233	R237	0.48	-0.13	No
T174Q/F196Y	4gmf	D	Q99	Y122	0.48	-0.119	No
I14W/T18Y	5std	C	W26	Y30	0.35	-0.1	No
V41R/K45N	3ix8	A	R137	N141	0.36	-0.052	No
T57N/N64S	3o41	L	N161	S177	0.49	-0.022	No
P183D/Y187W	4ggo	A	D205	W209	0.48	0.027	No
T18W/G22S	2eij	C	W146	S150	0.19	0.046	No
G111K/Q118K	3ly3	A	K277	K284	0.46	0.066	No
S114Q/Q118R	2x1c	B	Q196	R200	0.47	0.097	No

Mutation	Template	Chain	R1	R2	RMSD	ΔΔG	Clash
P183H/Y187W	4enq	D	H251	W255	0.5	0.133	No
T184K/S191R	4r0y	A	K827	R834	0.43	0.148	No
E129R/A133S	4ood	A	R31	S35	0.34	0.167	No
Y187W/S191Q	2q79	A	W339	Q343	0.3	0.195	No
A110T/S114Q	3c9x	A	T109	Q113	0.49	0.24	No
Q44Y/P48R	4q5e	C	Y129	R133	0.47	0.286	No
G111R/S115N	3ix8	A	R137	N141	0.45	0.294	No
A110S/S114W	3o4w	B	S123	W127	0.43	0.305	No
F97T/K107T	1bu8	A	T414	T437	0.5	0.312	No
V41S/K45W	3o4w	B	S123	W127	0.42	0.354	No
P183T/Y187R	3mux	A	T233	R237	0.26	0.459	No
T11R/Q15N	3ix8	A	R137	N141	0.28	0.469	No
P183T/Y187Q	3c9x	A	T109	Q113	0.39	0.504	No
S114H/Q118W	4enq	D	H251	W255	0.35	0.512	No
Y116W/V120Q	1uza	A	W104	Q108	0.38	0.516	No
S114E/Q118Y	3ub9	A	E48	Y52	0.36	0.525	No
K226D/Q233E	4gqn	A	D13	E27	0.45	0.635	No
S114K/Q118H	2ifq	A	K39	H43	0.47	0.643	No
S114Q/Q118Y	1c27	A	Q130	Y134	0.46	0.645	No
Q15R/K19R	1qtx	B	R12	R16	0.31	0.675	No
G111K/S115N	3lk4	W	K237	N241	0.28	0.716	No

Mutation	Template	Chain	R1	R2	RMSD	ΔΔG	Clash
Q44K/P48N	4nat	A	K108	N112	0.5	0.722	No
P183S/Y187D	4iqg	B	S37	D41	0.47	0.759	No
Y187W/S191T	1c5v	A	W237	T241	0.4	0.794	No
S114H/Q118Y	1xh8	A	H142	Y146	0.4	0.805	No
Y187D/S191W	4ggo	A	D205	W209	0.35	0.815	No
G111H/S115N	4hmm	B	H177	N181	0.29	0.833	No
P183K/Y187H	2ifq	A	K39	H43	0.48	0.847	No
A110S/S114D	4iqg	B	S37	D41	0.35	0.864	No
S114H/Q118N	4hmm	B	H177	N181	0.46	0.876	No
S114K/Q118N	3lk4	W	K237	N241	0.27	0.965	No
P183T/Y187S	3hh1	C	T43	S47	0.42	0.968	No
P183R/Y187N	3ix8	A	R137	N141	0.17	0.995	No
P125N/E129Y	3vlw	B	N375	Y379	0.23	1.001	No
A12Q/E16H	2gj3	A	Q29	H33	0.49	1.02	No
G111N/S115T	3bol	B	N190	T194	0.4	1.02	No
L113W/E117W	4tf4	A	W287	W291	0.39	1.069	No
Y187S/S191K	4kn8	B	S26	K30	0.33	1.084	No
S114H/Q118S	1p7g	H	H87	S91	0.45	1.092	No
Y66W/V79Q	4h3l	A	W93	Q109	0.33	1.178	No
L113H/E117W	4enq	D	H251	W255	0.34	1.234	No
A8S/I14R	4i7e	B	S255	R261	0.42	1.251	No

Mutation	Template	Chain	R1	R2	RMSD	ΔΔG	Clash
G111S/S115H	4toh	B	S126	H130	0.21	1.253	No
R69S/I76Y	1k8k	D	S166	Y222	0.48	1.28	No
D157R/R161S	4ood	A	R31	S35	0.39	1.288	No
P125S/E129K	4kn8	B	S26	K30	0.16	1.293	No
P183S/Y187K	4kn8	B	S26	K30	0.49	1.293	No
T18H/G22S	1p7g	H	H87	S91	0.35	1.3	No
P183H/Y187N	4hmm	B	H177	N181	0.47	1.303	No
G111S/S115K	4kn8	B	S26	K30	0.23	1.309	No
T174S/F196H	8dfr	A	S6	H131	0.45	1.33	No
Q15N/K19T	3bol	B	N190	T194	0.46	1.331	No
L113H/E117Y	1xh8	A	H142	Y146	0.37	1.371	No
T11S/Q15W	3o4w	B	S123	W127	0.39	1.376	No
T174E/F196H	3apt	A	E18	H270	0.45	1.409	No
L113Q/E117Y	1c27	A	Q130	Y134	0.27	1.41	No
L113Q/E117H	2gj3	A	Q29	H33	0.5	1.417	No
L113K/E117H	2ifq	A	K39	H43	0.31	1.421	No
P125S/E129H	4toh	B	S126	H130	0.24	1.454	No
V221S/I234Q	5a51	B	S154	Q165	0.43	1.477	No
V120S/F124S	1pbw	B	S265	S269	0.47	1.49	No
L113N/E117T	3bol	B	N190	T194	0.45	1.512	No
L113T/E117H	3vpc	C	T148	H152	0.28	1.52	No

Mutation	Template	Chain	R1	R2	RMSD	ΔΔG	Clash
Y66W/V79N	2wht	C	W89	N105	0.39	1.521	No
L113E/E117Y	3ub9	A	E48	Y52	0.22	1.526	No
L113S/E117H	4toh	B	S126	H130	0.26	1.561	No
I14W/T18S	2eij	C	W146	S150	0.2	1.58	No
L113D/E117W	4ggo	A	D205	W209	0.23	1.598	No
T184S/Y188D	4iqg	B	S37	D41	0.49	1.653	No
V67Y/I76S	2zws	A	Y148	S183	0.39	1.671	No
F124T/Q128S	3hh1	C	T43	S47	0.46	1.683	No
L131K/P135N	4nat	A	K108	N112	0.44	1.754	No
F124S/Q128D	4iqg	B	S37	D41	0.38	1.775	No
T184N/Y188T	3bol	B	N190	T194	0.42	1.807	No
Q13H/F17S	1p7g	H	H87	S91	0.35	1.816	No
K19E/I23Y	3ub9	A	E48	Y52	0.16	1.849	No
F124N/Q128T	3bol	B	N190	T194	0.42	1.861	No
V67Y/I76N	4nbz	A	Y30	N37	0.47	1.905	No
L113H/E117N	4hmm	B	H177	N181	0.35	1.912	No
T184N/Y188E	4dno	B	N233	E237	0.42	1.971	No
F124Q/Q128Y	1c27	A	Q130	Y134	0.37	1.995	No
V126T/Q130H	3vpc	C	T148	H152	0.17	2.025	No
F124Q/Q128R	2x1c	B	Q196	R200	0.31	2.027	No
Q13H/F17N	4hmm	B	H177	N181	0.35	2.053	No

Mutation	Template	Chain	R1	R2	RMSD	ΔΔG	Clash
Q13N/F17T	3bol	B	N190	T194	0.39	2.093	No
F124Y/Q128T	4cae	C	Y88	T92	0.27	2.125	No
I14S/T18Q	2ao9	C	S96	Q100	0.23	2.127	No
A151N/G155T	3bol	B	N190	T194	0.31	2.156	No
K19Q/I23Y	1c27	A	Q130	Y134	0.22	2.168	No
F124E/Q128Y	3ub9	A	E48	Y52	0.31	2.17	No
V126N/Q130T	3bol	B	N190	T194	0.4	2.182	No
F124S/Q128H	4toh	B	S126	H130	0.38	2.213	No
M156N/Q160T	3bol	B	N190	T194	0.35	2.225	No
T184Q/Y188H	2gj3	A	Q29	H33	0.44	2.226	No
F124H/Q128N	4hmm	B	H177	N181	0.28	2.232	No
Q13E/F17Y	3ub9	A	E48	Y52	0.27	2.236	No
L113H/E117S	1p7g	H	H87	S91	0.34	2.237	No
F124W/Q128T	1c5v	A	W237	T241	0.35	2.246	No
Q13S/F17H	4toh	B	S126	H130	0.27	2.246	No
L113K/E117N	3lk4	W	K237	N241	0.24	2.249	No
L68Y/Y77N	4yal	B	Y478	N486	0.4	2.264	No
F185K/V189H	2ifq	A	K39	H43	0.3	2.287	No
Q13S/F17Y	3r3r	A	S159	Y163	0.5	2.288	No
A186N/E190T	3bol	B	N190	T194	0.4	2.289	No
F185Q/V189Y	1c27	A	Q130	Y134	0.25	2.29	No

Mutation	Template	Chain	R1	R2	RMSD	ΔΔG	Clash
F124H/Q128S	1p7g	H	H87	S91	0.45	2.299	No
T184H/Y188S	1p7g	H	H87	S91	0.4	2.309	No
F124K/L131R	4r0y	A	K827	R834	0.5	2.318	No
I14S/T18H	4toh	B	S126	H130	0.3	2.318	No
F86T/I90S	3hh1	C	T43	S47	0.38	2.319	No
G181K/F185H	4j6j	A	K182	H186	0.48	2.32	No
L153N/D157T	3bol	B	N190	T194	0.36	2.34	No
I14S/T18K	4kn8	B	S26	K30	0.22	2.37	No
V67T/I76H	3fe4	A	T214	H229	0.45	2.37	No
V126S/Q130D	4iqg	B	S37	D41	0.49	2.375	No
I14H/T18S	1p7g	H	H87	S91	0.46	2.376	No
F185Y/V189T	4cae	C	Y88	T92	0.26	2.439	No
L146S/Q150H	4toh	B	S126	H130	0.49	2.446	No
V126S/Q130H	4toh	B	S126	H130	0.22	2.449	No
F124N/Q128Y	3vlw	B	N375	Y379	0.34	2.45	No
I52N/Y66T	4emd	A	N23	T41	0.44	2.45	No
L68Q/V79T	1ajs	B	Q254	T271	0.33	2.463	No
A9D/F17R	1m22	B	D147	R155	0.35	2.472	No
A149Y/L153T	4cae	C	Y88	T92	0.32	2.509	No
F124R/Q128N	3ix8	A	R137	N141	0.44	2.536	No
R69S/I76T	3oh8	A	S6	T106	0.32	2.536	No

Mutation	Template	Chain	R1	R2	RMSD	ΔΔG	Clash
V127K/L131H	2ifq	A	K39	H43	0.3	2.541	No
I76H/V139Q	3oe1	A	H21	Q44	0.36	2.544	No
L152E/M156Y	3ub9	A	E48	Y52	0.23	2.611	No
V126S/Q130K	4kn8	B	S26	K30	0.22	2.634	No
K19H/I23K	1k3t	C	H349	K353	0.5	2.641	No
M156S/Q160K	4kn8	B	S26	K30	0.5	2.652	No
K19S/I23Y	3r3r	A	S159	Y163	0.49	2.661	No
V67H/I76K	5h8t	A	H116	K127	0.45	2.67	No
F56T/V67K	1naq	A	T41	K67	0.41	2.675	No
F185H/V189N	4hmm	B	H177	N181	0.26	2.68	No
F185N/V189T	3bol	B	N190	T194	0.4	2.682	No
F124K/Q128N	3lk4	W	K237	N241	0.38	2.683	No
F124K/Q128H	2ifq	A	K39	H43	0.2	2.696	No
A149S/L153Y	3r3r	A	S159	Y163	0.5	2.701	No
F185T/V189H	3vpc	C	T148	H152	0.18	2.702	No
V67Q/I76T	1h1i	B	Q83	T95	0.49	2.702	No
A149S/L153K	4kn8	B	S26	K30	0.24	2.711	No
K19N/I23T	3bol	B	N190	T194	0.4	2.712	No
F124S/Q128K	4kn8	B	S26	K30	0.27	2.738	No
K19N/I23E	4dno	B	N233	E237	0.49	2.741	No
L152N/M156Y	3vlw	B	N375	Y379	0.31	2.745	No

Mutation	Template	Chain	R1	R2	RMSD	ΔΔG	Clash
F86S/I90D	4iqg	B	S37	D41	0.43	2.771	No
I52S/Y66H	3f5o	B	S83	H134	0.46	2.798	No
Q13S/F17K	4kn8	B	S26	K30	0.24	2.8	No
L152Q/M156H	2gj3	A	Q29	H33	0.43	2.802	No
K19H/I23Y	1xh8	A	H142	Y146	0.28	2.812	No
F124T/Q128R	3mux	A	T233	R237	0.43	2.845	No
A149S/L153H	4toh	B	S126	H130	0.3	2.851	No
A154S/L158H	4toh	B	S126	H130	0.29	2.854	No
A154Y/L158T	4cae	C	Y88	T92	0.28	2.881	No
F185S/V189H	4toh	B	S126	H130	0.22	2.889	No
F185K/V189N	3lk4	W	K237	N241	0.24	2.898	No
V127T/L131H	3vpc	C	T148	H152	0.22	2.912	No
K19S/I23H	4toh	B	S126	H130	0.2	2.921	No
F185H/V189S	1p7g	H	H87	S91	0.37	2.928	No
K19H/I23S	1p7g	H	H87	S91	0.47	2.955	No
I52D/Y66Q	3vc1	G	D248	Q283	0.38	2.981	No
L78T/V139K	5dzq	O	T135	K184	0.37	3.011	No
L152S/M156W	3o4w	B	S123	W127	0.49	3.026	No
Q42R/W46S	4ood	A	R31	S35	0.41	3.032	No
F185S/V189K	4kn8	B	S26	K30	0.24	3.035	No
V127H/L131N	4hmm	B	H177	N181	0.35	3.045	No

Mutation	Template	Chain	R1	R2	RMSD	ΔΔG	Clash
L78E/V139H	3apt	A	E18	H270	0.44	3.052	No
K19H/I23N	4hmm	B	H177	N181	0.25	3.063	No
V67T/I76N	3lw2	A	T93	N167	0.43	3.104	No
L78H/V139N	2cki	B	H72	N172	0.38	3.107	No
Y66Q/Y77R	3aeh	A	Q1247	R1282	0.5	3.117	No
V127N/L131Y	3vlw	B	N375	Y379	0.19	3.118	No
V127D/L131W	4ggo	A	D205	W209	0.18	3.16	No
I52D/Y66K	4ivd	B	D880	K888	0.46	3.21	No
Y77K/I140T	2yaf	A	K43	T83	0.32	3.211	No
V67Q/L78N	2jjo	A	Q292	N313	0.45	3.214	No
V67H/L78S	3bzn	A	H223	S237	0.41	3.229	No
I52T/Y66E	3ot8	A	T14	E22	0.4	3.27	No
V127H/L131S	1p7g	H	H87	S91	0.32	3.292	No
V67D/I76K	1gd1	R	D241	K306	0.4	3.295	No
L152H/M156N	4hmm	B	H177	N181	0.23	3.297	No
L152S/M156H	4toh	B	S126	H130	0.32	3.298	No
L152N/M156T	3bol	B	N190	T194	0.29	3.329	No
L152H/M156S	1p7g	H	H87	S91	0.38	3.364	No
V127S/L131H	4toh	B	S126	H130	0.17	3.388	No
Y66H/Y77N	4d5d	B	H24	N55	0.43	3.414	No
A154S/L158D	4iqg	B	S37	D41	0.5	3.437	No

Mutation	Template	Chain	R1	R2	RMSD	ΔΔG	Clash
V127N/L131T	3bol	B	N190	T194	0.45	3.477	No
V67N/L78S	3o41	L	N161	S177	0.33	3.579	No
V67S/L78T	3oh8	A	S6	T106	0.38	3.647	No
L68S/Y77H	3nrq	B	S71	H115	0.39	3.65	No
I52S/Y66E	1ra1	A	S135	E154	0.36	3.699	No

Showing 1 to 213 of 213 entries

Table S5. Three-hundred forty-four mutants suggested for the β-glucosidase (hydrolase; 1BGA).

Proteus ID: 4L8HLU. Available at <http://proteus.dcc.ufmg.br/result/id/4L8HLU>.

Mutation	Template	Chain	R1	R2	RMSD	ΔΔG	Clash
V264W/A268S	2eij	C	W146	S150	0.16	-0.83	No
R207Q/L211Q	3n0y	A	Q26	Q30	0.21	-0.762	No
V264W/A268T	1c5v	A	W237	T241	0.32	-0.734	No
R302H/I315N	5f9o	H	H35	N99	0.5	-0.729	No
V380E/R384Y	3ub9	A	E48	Y52	0.16	-0.705	No
V264H/A268N	4hmm	B	H177	N181	0.24	-0.66	No
R138Y/A142T	4cae	C	Y88	T92	0.49	-0.657	No
V264H/A268S	1p7g	H	H87	S91	0.34	-0.633	No
R138D/A142Q	2rin	B	D251	Q255	0.41	-0.628	No
V380Q/R384Y	1c27	A	Q130	Y134	0.16	-0.606	No
V264Y/A268T	4cae	C	Y88	T92	0.23	-0.598	No
V380W/R384S	2eij	C	W146	S150	0.1	-0.555	No
V264Q/A268Y	1c27	A	Q130	Y134	0.25	-0.545	No
H383N/H387T	3bol	B	N190	T194	0.44	-0.518	No
V380H/R384N	4hmm	B	H177	N181	0.24	-0.505	No
R103S/L107H	4toh	B	S126	H130	0.39	-0.483	No
V264Q/A268R	2x1c	B	Q196	R200	0.23	-0.48	No
V380D/R384W	4ggo	A	D205	W209	0.18	-0.48	No
V380N/R384E	4dno	B	N233	E237	0.39	-0.47	No
V264N/A268T	3bol	B	N190	T194	0.4	-0.469	No

Mutation	Template	Chain	R1	R2	RMSD	ΔΔG	Clash
V380H/R384W	4enq	D	H251	W255	0.33	-0.462	No
V380H/R384Y	1xh8	A	H142	Y146	0.33	-0.459	No
V264R/A268N	3ix8	A	R137	N141	0.5	-0.437	No
V380Y/R384T	4cae	C	Y88	T92	0.25	-0.436	No
V105N/N109E	4dno	B	N233	E237	0.49	-0.421	No
S373E/Q377Y	3ub9	A	E48	Y52	0.18	-0.404	No
S373R/Q377W	1w35	A	R163	W167	0.31	-0.4	No
V380S/R384Y	3r3r	A	S159	Y163	0.44	-0.397	No
V205S/R209H	4toh	B	S126	H130	0.23	-0.387	No
A237H/R241N	4hmm	B	H177	N181	0.29	-0.367	No
S373N/Q377Y	3vlw	B	N375	Y379	0.23	-0.353	No
A240N/S244T	3bol	B	N190	T194	0.39	-0.348	No
L98N/H102E	4dno	B	N233	E237	0.32	-0.326	No
S373Q/Q377Y	1c27	A	Q130	Y134	0.31	-0.313	No
L98D/H102Q	2rin	B	D251	Q255	0.31	-0.312	No
V380N/R384T	3bol	B	N190	T194	0.4	-0.296	No
V144D/E148N	4nac	B	D65	N69	0.46	-0.276	No
D192N/H196E	4dno	B	N233	E237	0.42	-0.275	No
V104E/L108Y	3ub9	A	E48	Y52	0.2	-0.268	No
V380H/R384S	1p7g	H	H87	S91	0.41	-0.267	No
Q141D/Q145W	4ggo	A	D205	W209	0.14	-0.266	No
K236R/A240R	1qtx	B	R12	R16	0.23	-0.26	No

Mutation	Template	Chain	R1	R2	RMSD	ΔΔG	Clash
V105S/N109D	4iqg	B	S37	D41	0.5	-0.251	No
V380S/R384H	4toh	B	S126	H130	0.18	-0.244	No
H195N/V199Y	3vlw	B	N375	Y379	0.24	-0.24	No
Y334N/H338E	4dno	B	N233	E237	0.5	-0.224	No
Y100D/V104W	4ggo	A	D205	W209	0.28	-0.218	No
Q141R/Q145W	1w35	A	R163	W167	0.36	-0.196	No
F78W/P115T	2yp6	C	W77	T106	0.47	-0.193	No
V264S/A268K	4kn8	B	S26	K30	0.16	-0.177	No
I65N/K69T	3bol	B	N190	T194	0.43	-0.171	No
V199E/L203Y	3ub9	A	E48	Y52	0.17	-0.159	No
Q381N/T385E	4dno	B	N233	E237	0.44	-0.158	No
L107Q/N111Q	3n0y	A	Q26	Q30	0.32	-0.145	No
Y334D/H338N	4nac	B	D65	N69	0.47	-0.141	No
T37R/T41S	3cvo	D	R196	S200	0.43	-0.13	No
V380K/R384H	2ifq	A	K39	H43	0.28	-0.123	No
W434D/V438Q	2rin	B	D251	Q255	0.34	-0.122	No
D192N/H196T	3bol	B	N190	T194	0.28	-0.118	No
H195N/V199E	4dno	B	N233	E237	0.45	-0.115	No
V380S/R384K	4kn8	B	S26	K30	0.19	-0.115	No
Q188N/D192E	4dno	B	N233	E237	0.43	-0.114	No
I3E/E445N	3sys	A	E343	N369	0.4	-0.106	No
Q141N/Q145E	4dno	B	N233	E237	0.46	-0.105	No

Mutation	Template	Chain	R1	R2	RMSD	ΔΔG	Clash
W434N/V438Y	3vlw	B	N375	Y379	0.33	-0.104	No
V205K/R209N	3lk4	W	K237	N241	0.31	-0.098	No
E233S/A237Q	2ao9	C	S96	Q100	0.47	-0.097	No
G279S/I283R	4ise	A	S418	R422	0.26	-0.082	No
L203N/R207T	3bol	B	N190	T194	0.46	-0.082	No
F143Q/A147Y	1c27	A	Q130	Y134	0.15	-0.08	No
W434Y/V438Y	3i0z	A	Y292	Y296	0.5	-0.073	No
V321E/G325S	2z84	A	E163	S167	0.35	-0.069	No
H201N/V205T	3bol	B	N190	T194	0.45	-0.05	No
S373N/Q377T	3bol	B	N190	T194	0.36	-0.046	No
Y101Q/V105Y	1c27	A	Q130	Y134	0.3	-0.042	No
V144S/E148D	4iqg	B	S37	D41	0.47	-0.04	No
V199Q/L203Y	1c27	A	Q130	Y134	0.18	-0.04	No
V199Y/L203T	4cae	C	Y88	T92	0.23	-0.038	No
R371Y/M375T	4cae	C	Y88	T92	0.26	-0.033	No
Y334N/H338T	3bol	B	N190	T194	0.32	-0.028	No
D99N/R103T	3bol	B	N190	T194	0.46	-0.024	No
S373Q/Q377R	2x1c	B	Q196	R200	0.25	-0.015	No
D369R/S373R	1qtx	B	R12	R16	0.47	-0.008	No
Q376N/V380T	3bol	B	N190	T194	0.5	-0.005	No
E233Q/A237R	2x1c	B	Q196	R200	0.49	0.023	No
V264S/A268H	4toh	B	S126	H130	0.25	0.023	No

Mutation	Template	Chain	R1	R2	RMSD	ΔΔG	Clash
I3Q/E445N	2jjo	A	Q292	N313	0.48	0.031	No
W434S/V438Y	3r3r	A	S159	Y163	0.44	0.031	No
F146W/M150W	4tf4	A	W287	W291	0.38	0.033	No
H195S/V199Y	3r3r	A	S159	Y163	0.48	0.038	No
I140N/V144E	4dno	B	N233	E237	0.42	0.038	No
V321T/G325D	5df7	B	T168	D172	0.44	0.049	No
T189N/V193T	3bol	B	N190	T194	0.35	0.069	No
H195N/V199T	3bol	B	N190	T194	0.4	0.07	No
V380K/H387R	4r0y	A	K827	R834	0.48	0.071	No
Y334H/H338W	4enq	D	H251	W255	0.28	0.084	No
W434Y/V438T	4cae	C	Y88	T92	0.33	0.099	No
I3N/E445S	3o41	L	N161	S177	0.32	0.104	No
V199H/L203W	4enq	D	H251	W255	0.31	0.107	No
Q376S/V380D	4iqg	B	S37	D41	0.5	0.114	No
H102N/D106T	3bol	B	N190	T194	0.32	0.124	No
I140S/V144Q	2ao9	C	S96	Q100	0.11	0.126	No
Y100S/V104Y	3r3r	A	S159	Y163	0.49	0.132	No
A147N/F151E	4dno	B	N233	E237	0.39	0.137	No
Y61N/I65Y	3vlw	B	N375	Y379	0.46	0.14	No
V205N/R209T	3bol	B	N190	T194	0.34	0.142	No
L124D/Q129E	4cku	E	D137	E142	0.4	0.146	No
Y433W/N437T	1c5v	A	W237	T241	0.44	0.146	No

Mutation	Template	Chain	R1	R2	RMSD	ΔΔG	Clash
G332S/V336D	4iqg	B	S37	D41	0.42	0.153	No
A147S/F151Y	3r3r	A	S159	Y163	0.43	0.167	No
V199N/L203Y	3vlw	B	N375	Y379	0.25	0.172	No
L173E/V179N	4jzk	B	E185	N190	0.45	0.175	No
V104N/L108Y	3vlw	B	N375	Y379	0.3	0.182	No
Y61N/I65E	4dno	B	N233	E237	0.47	0.183	No
T189H/V193S	1p7g	H	H87	S91	0.4	0.188	No
Y432S/R436Q	2ao9	C	S96	Q100	0.23	0.191	No
F143S/A147Y	3r3r	A	S159	Y163	0.43	0.194	No
S373H/Q377W	4enq	D	H251	W255	0.27	0.201	No
Y101N/V105E	4dno	B	N233	E237	0.48	0.203	No
V199S/L203Y	3r3r	A	S159	Y163	0.48	0.207	No
Y374S/H378Y	3r3r	A	S159	Y163	0.45	0.213	No
Q141N/Q145T	3bol	B	N190	T194	0.43	0.22	No
V93N/L98S	4zty	A	N304	S309	0.49	0.221	No
Y334H/H338N	4hmm	B	H177	N181	0.17	0.227	No
S373K/Q377H	2ifq	A	K39	H43	0.27	0.236	No
Y374H/H378N	4hmm	B	H177	N181	0.23	0.247	No
H378T/V382H	3vpc	C	T148	H152	0.22	0.263	No
I3S/E445T	3oh8	A	S6	T106	0.33	0.265	No
W434N/V438T	3bol	B	N190	T194	0.39	0.266	No
I372N/Q376T	3bol	B	N190	T194	0.35	0.274	No

Mutation	Template	Chain	R1	R2	RMSD	ΔΔG	Clash
E148N/R152T	3bol	B	N190	T194	0.19	0.278	No
G97T/Y101H	3vpc	C	T148	H152	0.28	0.279	No
V144N/E148T	3bol	B	N190	T194	0.35	0.279	No
Y435S/V439Y	3r3r	A	S159	Y163	0.47	0.286	No
V419S/R426Y	1k8k	D	S166	Y222	0.46	0.288	No
V199N/L203E	4dno	B	N233	E237	0.46	0.294	No
Y334H/H338S	1p7g	H	H87	S91	0.42	0.296	No
Y374N/H378T	3bol	B	N190	T194	0.32	0.299	No
M290D/Y349W	1xtb	A	D150	W181	0.48	0.308	No
S373H/Q377Y	1xh8	A	H142	Y146	0.29	0.309	No
D265R/E269R	1qtx	B	R12	R16	0.37	0.318	No
W434H/V438N	4hmm	B	H177	N181	0.3	0.32	No
G194T/L198R	3mux	A	T233	R237	0.49	0.321	No
I140S/V144D	4iqg	B	S37	D41	0.49	0.324	No
E233W/A237Y	5std	C	W26	Y30	0.47	0.338	No
V105N/N109T	3bol	B	N190	T194	0.32	0.338	No
L187T/I191Q	3c9x	A	T109	Q113	0.46	0.346	No
T149H/E153N	4hmm	B	H177	N181	0.43	0.346	No
Y101S/V105Y	3r3r	A	S159	Y163	0.47	0.351	No
V419E/Q424N	2gop	B	E48	N53	0.38	0.355	No
T149N/E153T	3bol	B	N190	T194	0.24	0.358	No
D235N/C239T	3bol	B	N190	T194	0.42	0.363	No

Mutation	Template	Chain	R1	R2	RMSD	ΔΔG	Clash
W12S/Y395W	5cgm	A	S228	W526	0.48	0.366	No
G194S/L198H	4toh	B	S126	H130	0.26	0.369	No
Q188N/D192T	3bol	B	N190	T194	0.39	0.369	No
C239N/I243T	3bol	B	N190	T194	0.21	0.371	No
I191N/H195T	3bol	B	N190	T194	0.24	0.381	No
I372S/Q376H	4toh	B	S126	H130	0.31	0.386	No
F146Y/M150T	4cae	C	Y88	T92	0.28	0.398	No
E233S/A237K	4kn8	B	S26	K30	0.46	0.399	No
I140N/V144T	3bol	B	N190	T194	0.45	0.404	No
F172T/M176N	3vc8	B	T65	N69	0.46	0.414	No
R371H/M375N	4hmm	B	H177	N181	0.19	0.445	No
H195S/V199H	4toh	B	S126	H130	0.17	0.463	No
S373K/Q377N	3lk4	W	K237	N241	0.3	0.472	No
H201T/V205H	3vpc	C	T148	H152	0.25	0.475	No
Y432S/R436K	4kn8	B	S26	K30	0.25	0.475	No
C169S/L173K	2fv0	B	S94	K98	0.35	0.483	No
E233T/A237Q	3c9x	A	T109	Q113	0.43	0.494	No
V199H/L203N	4hmm	B	H177	N181	0.19	0.496	No
V321T/G325R	2v3v	A	T704	R709	0.41	0.518	No
E233W/A237S	2eij	C	W146	S150	0.49	0.524	No
I291K/I348Y	3n29	A	K35	Y231	0.45	0.524	No
Y76E/I113W	2bwa	A	E124	W161	0.5	0.538	No

Mutation	Template	Chain	R1	R2	RMSD	ΔΔG	Clash
I3N/W443D	1aoz	B	N228	D253	0.35	0.539	No
W434S/V438H	4toh	B	S126	H130	0.27	0.546	No
G97S/Y101H	4toh	B	S126	H130	0.26	0.574	No
L333Y/L337T	4cae	C	Y88	T92	0.22	0.574	No
L263Q/F267H	2gj3	A	Q29	H33	0.4	0.576	No
Y334N/H338K	1k0n	A	N179	K183	0.49	0.579	No
Q188R/D192N	3ix8	A	R137	N141	0.48	0.583	No
V199Q/L203R	2x1c	B	Q196	R200	0.18	0.591	No
F143Q/A147R	2x1c	B	Q196	R200	0.16	0.615	No
H201S/V205H	4toh	B	S126	H130	0.25	0.616	No
A147D/F151N	4nac	B	D65	N69	0.43	0.619	No
V382N/I386T	3bol	B	N190	T194	0.36	0.621	No
Y435H/V439N	4hmm	B	H177	N181	0.35	0.627	No
V199N/L203T	3bol	B	N190	T194	0.28	0.635	No
L333D/L337W	4ggo	A	D205	W209	0.23	0.637	No
V144H/E148N	4hmm	B	H177	N181	0.21	0.637	No
W434Q/V438R	2x1c	B	Q196	R200	0.32	0.641	No
V104H/L108N	4hmm	B	H177	N181	0.17	0.642	No
H195K/V199N	3lk4	W	K237	N241	0.3	0.654	No
H378S/V382H	4toh	B	S126	H130	0.14	0.654	No
V93S/L98K	1g8k	C	S809	K814	0.36	0.654	No
F146W/M150S	2eij	C	W146	S150	0.2	0.663	No

Mutation	Template	Chain	R1	R2	RMSD	ΔΔG	Clash
E62N/R66T	3bol	B	N190	T194	0.39	0.664	No
A147H/F151N	4hmm	B	H177	N181	0.23	0.665	No
F146H/M150Y	1xh8	A	H142	Y146	0.18	0.671	No
Y432H/R436S	1p7g	H	H87	S91	0.31	0.682	No
Y435N/V439T	3bol	B	N190	T194	0.29	0.688	No
Y435Q/V439K	4a9o	C	Q80	K84	0.43	0.694	No
D280S/I284D	4iqg	B	S37	D41	0.44	0.7	No
V419S/R426T	3oh8	A	S6	T106	0.47	0.705	No
T139N/F143T	3bol	B	N190	T194	0.44	0.706	No
E63N/L67T	3bol	B	N190	T194	0.35	0.712	No
A147S/F151H	4toh	B	S126	H130	0.25	0.714	No
H201S/V205K	4kn8	B	S26	K30	0.23	0.716	No
Y100H/V104N	4hmm	B	H177	N181	0.25	0.718	No
Y61H/I65N	4hmm	B	H177	N181	0.47	0.718	No
V199S/L203H	4toh	B	S126	H130	0.25	0.722	No
C239S/I243H	4toh	B	S126	H130	0.41	0.724	No
A147H/F151S	1p7g	H	H87	S91	0.38	0.738	No
V104N/L108E	4dno	B	N233	E237	0.47	0.738	No
I3W/E445S	2xgn	A	W91	S105	0.32	0.744	No
V382S/I386H	4toh	B	S126	H130	0.25	0.749	No
I372S/Q376K	4kn8	B	S26	K30	0.14	0.75	No
V193N/L197T	3bol	B	N190	T194	0.35	0.751	No

Mutation	Template	Chain	R1	R2	RMSD	ΔΔG	Clash
Y100N/V104T	3bol	B	N190	T194	0.34	0.76	No
F143N/A147T	3bol	B	N190	T194	0.37	0.763	No
R371S/M375H	4toh	B	S126	H130	0.3	0.766	No
Y374K/H378N	3lk4	W	K237	N241	0.32	0.78	No
W434K/V438N	3lk4	W	K237	N241	0.33	0.793	No
W434N/V438K	1k0n	A	N179	K183	0.49	0.797	No
V199D/L203N	4nac	B	D65	N69	0.44	0.799	No
H201K/V205H	2ifq	A	K39	H43	0.26	0.811	No
Y374H/H378S	1p7g	H	H87	S91	0.48	0.816	No
V199H/L203S	1p7g	H	H87	S91	0.43	0.82	No
A147S/F151D	4iqg	B	S37	D41	0.49	0.821	No
V419Q/R426N	2jjo	A	Q292	N313	0.45	0.825	No
Y334R/H338N	3ix8	A	R137	N141	0.45	0.825	No
Y61T/I65H	3vpc	C	T148	H152	0.45	0.825	No
L187T/I191S	3hh1	C	T43	S47	0.28	0.827	No
F4S/L444W	2bi0	A	S311	W330	0.36	0.833	No
R371D/M375N	4nac	B	D65	N69	0.4	0.839	No
V104K/L108H	2ifq	A	K39	H43	0.29	0.841	No
V104H/L108S	1p7g	H	H87	S91	0.39	0.844	No
V144H/E148S	1p7g	H	H87	S91	0.43	0.849	No
Y101N/V105T	3bol	B	N190	T194	0.32	0.853	No
H201S/V205D	4iqg	B	S37	D41	0.48	0.863	No

Mutation	Template	Chain	R1	R2	RMSD	ΔΔG	Clash
V104S/L108H	4toh	B	S126	H130	0.27	0.864	No
E233T/A237S	3hh1	C	T43	S47	0.34	0.865	No
F143H/A147S	1p7g	H	H87	S91	0.41	0.881	No
R371H/M375S	1p7g	H	H87	S91	0.43	0.883	No
E233H/A237N	4hmm	B	H177	N181	0.45	0.891	No
V104T/L108R	3mux	A	T233	R237	0.5	0.895	No
V199R/L203N	3ix8	A	R137	N141	0.48	0.9	No
E148N/R152Y	3vlw	B	N375	Y379	0.4	0.91	No
I293H/I348T	3l6d	B	H143	T164	0.36	0.913	No
Y61S/I65D	4iqg	B	S37	D41	0.25	0.918	No
V419N/R426S	3o41	L	N161	S177	0.47	0.926	No
V104N/L108T	3bol	B	N190	T194	0.35	0.928	No
V193S/L197H	4toh	B	S126	H130	0.33	0.932	No
Y334S/H338K	4kn8	B	S26	K30	0.17	0.938	No
V199K/L203H	2ifq	A	K39	H43	0.3	0.941	No
F172N/I191R	1t2b	A	N146	R245	0.42	0.944	No
V193H/L197N	4hmm	B	H177	N181	0.22	0.946	No
Y61S/I65H	4toh	B	S126	H130	0.48	0.946	No
Y100S/V104H	4toh	B	S126	H130	0.26	0.951	No
R371N/M375T	3bol	B	N190	T194	0.3	0.954	No
M375N/L379E	4dno	B	N233	E237	0.28	0.957	No
V104D/L108N	4nac	B	D65	N69	0.49	0.962	No

Mutation	Template	Chain	R1	R2	RMSD	ΔΔG	Clash
V336T/L340H	3vpc	C	T148	H152	0.4	0.976	No
Y435H/V439S	1p7g	H	H87	S91	0.49	0.978	No
I140T/V199K	2prb	A	T120	K156	0.48	0.998	No
F143S/A147D	4iqg	B	S37	D41	0.49	1.026	No
Y433S/N437K	4kn8	B	S26	K30	0.33	1.035	No
D280S/I284H	4toh	B	S126	H130	0.35	1.049	No
W434S/V438K	4kn8	B	S26	K30	0.34	1.069	No
E233T/A237R	3mux	A	T233	R237	0.19	1.071	No
R371S/M375K	4kn8	B	S26	K30	0.15	1.075	No
Y374S/H378K	4kn8	B	S26	K30	0.23	1.093	No
F143K/A147N	3lk4	W	K237	N241	0.29	1.104	No
V199S/L203K	4kn8	B	S26	K30	0.15	1.107	No
Y374R/H378N	3ix8	A	R137	N141	0.49	1.11	No
Y61T/I65S	3hh1	C	T43	S47	0.41	1.117	No
L187K/I191H	2ifq	A	K39	H43	0.42	1.13	No
I140S/V199D	4nac	B	S27	D65	0.48	1.141	No
L263D/F267N	4nac	B	D65	N69	0.48	1.152	No
L263S/F267H	4toh	B	S126	H130	0.27	1.157	No
Y101S/V105H	4toh	B	S126	H130	0.39	1.166	No
A147S/F151K	4kn8	B	S26	K30	0.16	1.17	No
R241S/L245T	3a5s	B	S277	T281	0.46	1.171	No
V193H/L197S	1p7g	H	H87	S91	0.37	1.182	No

Mutation	Template	Chain	R1	R2	RMSD	ΔΔG	Clash
Y100K/V104N	3lk4	W	K237	N241	0.34	1.195	No
S373H/Q377S	1p7g	H	H87	S91	0.37	1.197	No
I293S/I348H	8dfr	A	S6	H131	0.49	1.213	No
W434K/V438H	2ifq	A	K39	H43	0.4	1.215	No
F146H/M150N	4hmm	B	H177	N181	0.18	1.218	No
Y435K/V439H	2ifq	A	K39	H43	0.39	1.238	No
Y435H/V439K	1k3t	C	H349	K353	0.48	1.25	No
Q95R/D99N	3ix8	A	R137	N141	0.36	1.251	No
L251K/Y255T	4ds4	D	K582	T586	0.39	1.267	No
L108D/I113N	4k38	B	D228	N233	0.43	1.275	No
V104S/L108D	4iqg	B	S37	D41	0.5	1.284	No
F146R/M150N	3ix8	A	R137	N141	0.48	1.304	No
Y100K/V104H	2ifq	A	K39	H43	0.34	1.31	No
Y100S/V104K	4kn8	B	S26	K30	0.3	1.31	No
D347R/K393R	4rpo	B	R195	R225	0.38	1.329	No
Y374H/H378K	1k3t	C	H349	K353	0.42	1.329	No
F143S/A147K	4kn8	B	S26	K30	0.21	1.342	No
Y61T/I65R	3mux	A	T233	R237	0.48	1.347	No
L333K/L337H	2ifq	A	K39	H43	0.3	1.351	No
V336S/L340H	4toh	B	S126	H130	0.39	1.356	No
Q261R/D265N	3ix8	A	R137	N141	0.26	1.359	No
L333N/L337E	4dno	B	N233	E237	0.47	1.367	No

Mutation	Template	Chain	R1	R2	RMSD	ΔΔG	Clash
D280T/I284R	3mux	A	T233	R237	0.49	1.392	No
L333H/L337N	4hmm	B	H177	N181	0.19	1.402	No
M375T/L379H	3vpc	C	T148	H152	0.27	1.41	No
L187S/I191D	4iqg	B	S37	D41	0.33	1.42	No
V193S/L197D	4iqg	B	S37	D41	0.45	1.424	No
E335R/Y339H	4xv8	A	R48	H52	0.15	1.43	No
Y61S/I65K	4kn8	B	S26	K30	0.4	1.437	No
L333N/L337T	3bol	B	N190	T194	0.35	1.46	No
L333R/L337N	3ix8	A	R137	N141	0.44	1.475	No
L333H/L337S	1p7g	H	H87	S91	0.35	1.513	No
F146D/M150N	4nac	B	D65	N69	0.48	1.519	No
L263K/F267N	3lk4	W	K237	N241	0.32	1.524	No
M375S/L379H	4toh	B	S126	H130	0.31	1.55	No
M375D/L379N	4nac	B	D65	N69	0.46	1.554	No
F146K/M150H	2ifq	A	K39	H43	0.29	1.557	No
L333S/L337H	4toh	B	S126	H130	0.34	1.558	No
I293D/I348T	1qvj	A	D172	T282	0.41	1.582	No
L333K/L337N	3lk4	W	K237	N241	0.33	1.586	No
I293E/I348N	1p3j	A	E114	N194	0.45	1.596	No
L263S/F267K	4kn8	B	S26	K30	0.2	1.603	No
F146H/M150S	1p7g	H	H87	S91	0.45	1.608	No
V336K/L340N	3lk4	W	K237	N241	0.41	1.615	No

Mutation	Template	Chain	R1	R2	RMSD	ΔΔG	Clash
Y101S/V105K	4kn8	B	S26	K30	0.26	1.641	No
F146N/M150T	3bol	B	N190	T194	0.3	1.658	No
F146S/M150H	4toh	B	S126	H130	0.26	1.666	No
V336S/L340K	4kn8	B	S26	K30	0.37	1.675	No
I348D/V392D	2fem	A	D148	D203	0.4	1.716	No
L333D/L337N	4nac	B	D65	N69	0.46	1.738	No
F6R/F10Y	3vaj	B	R228	Y232	0.34	1.739	No
M375S/L379D	4iqg	B	S37	D41	0.44	1.75	No
D106N/D110T	3bol	B	N190	T194	0.38	1.786	No
L333S/L337D	4iqg	B	S37	D41	0.47	1.854	No
I293E/I348D	4nu0	A	E115	D190	0.46	1.887	No
M375S/L379K	4kn8	B	S26	K30	0.21	2.013	No
E63H/L67N	4hmm	B	H177	N181	0.27	2.039	No
F146S/M150K	4kn8	B	S26	K30	0.21	2.048	No
L333S/L337K	4kn8	B	S26	K30	0.13	2.075	No
W266K/Q270R	1s2w	A	K102	R106	0.36	2.212	No

Showing 1 to 344 of 344 entries

Table S6. Two-hundred seventy-two mutants suggested for the lysozyme (2LZM) used in the second case study.

Proteus ID: SYLX52. Available at <http://proteus.dcc.ufmg.br/result/id/ SYLX52>.

Mutation	Template	Chain	R1	R2	RMSD	ΔΔG	Clash
D127Q/V131R	2x1c	B	Q196	R200	0.31	-1.506	No
D127T/V131R	3mux	A	T233	R237	0.5	-1.461	No
D127R/V131R	1qtx	B	R12	R16	0.24	-1.287	No
D127R/V131N	3ix8	A	R137	N141	0.48	-1.234	No
D127W/V131Q	2q79	A	W339	Q343	0.27	-1.215	No
K147R/T151R	1qtx	B	R12	R16	0.31	-1.181	No
D127S/V131K	4kn8	B	S26	K30	0.27	-1.165	No
D127H/V131N	4hmm	B	H177	N181	0.24	-1.023	No
D127W/V131S	2eij	C	W146	S150	0.25	-0.947	No
T109K/G113N	3lk4	W	K237	N241	0.47	-0.923	No
D127W/V131W	4tf4	A	W287	W291	0.34	-0.785	No
D70K/G77R	4r0y	A	K827	R834	0.32	-0.768	No
K147Q/T151Y	1c27	A	Q130	Y134	0.32	-0.751	No
D127Y/V131Y	3i0z	A	Y292	Y296	0.45	-0.72	No
D72N/R76E	4dno	B	N233	E237	0.46	-0.69	No
K147Q/T151R	2x1c	B	Q196	R200	0.22	-0.666	No
D127W/V131T	1c5v	A	W237	T241	0.39	-0.665	No
D127H/V131S	1p7g	H	H87	S91	0.46	-0.605	No
D127K/V131H	2ifq	A	K39	H43	0.34	-0.578	No
D127Y/V131T	4cae	C	Y88	T92	0.32	-0.564	No

Mutation	Template	Chain	R1	R2	RMSD	ΔΔG	Clash
A73Q/G77R	2x1c	B	Q196	R200	0.14	-0.552	No
D61R/K65R	1qtx	B	R12	R16	0.25	-0.518	No
A73R/G77R	1qtx	B	R12	R16	0.3	-0.485	No
K85Q/D89R	2x1c	B	Q196	R200	0.23	-0.452	No
D70K/G77K	3ly3	A	K277	K284	0.45	-0.433	No
S44K/K48N	3lk4	W	K237	N241	0.25	-0.432	No
D127Q/V131Y	1c27	A	Q130	Y134	0.28	-0.431	No
K43Q/D47R	2x1c	B	Q196	R200	0.22	-0.418	No
K147E/T151Y	3ub9	A	E48	Y52	0.23	-0.415	No
K43Q/D47Y	1c27	A	Q130	Y134	0.2	-0.415	No
D127H/V131Y	1xh8	A	H142	Y146	0.19	-0.41	No
N40R/S44W	1w35	A	R163	W167	0.32	-0.41	No
D127E/V131Y	3ub9	A	E48	Y52	0.16	-0.358	No
K85R/D89N	3ix8	A	R137	N141	0.46	-0.312	No
D127H/V131W	4enq	D	H251	W255	0.2	-0.308	No
T109E/G113Y	3ub9	A	E48	Y52	0.47	-0.293	No
P86Q/S90H	2gj3	A	Q29	H33	0.39	-0.291	No
A73S/G77K	4kn8	B	S26	K30	0.17	-0.246	No
K147H/T151W	4enq	D	H251	W255	0.4	-0.24	No
S44Q/K48Y	1c27	A	Q130	Y134	0.21	-0.201	No
N40R/S44N	3ix8	A	R137	N141	0.47	-0.164	No
K147H/T151Y	1xh8	A	H142	Y146	0.4	-0.163	No

Mutation	Template	Chain	R1	R2	RMSD	ΔΔG	Clash
A41Q/E45R	2x1c	B	Q196	R200	0.2	-0.158	No
K147D/T151W	4ggo	A	D205	W209	0.19	-0.147	No
D72K/L79K	3ly3	A	K277	K284	0.47	-0.115	No
T109D/G113W	4ggo	A	D205	W209	0.47	-0.074	No
K85N/D89E	4dno	B	N233	E237	0.45	-0.026	No
T115K/R119H	2ifq	A	K39	H43	0.4	-0.01	No
D127T/V131H	3vpc	C	T148	H152	0.2	0.0	No
T115R/R119N	3ix8	A	R137	N141	0.45	0.035	No
N68K/V75K	3ly3	A	K277	K284	0.42	0.041	No
T109W/G113W	4tf4	A	W287	W291	0.48	0.083	No
P143T/K147S	3hh1	C	T43	S47	0.21	0.114	No
P143R/K147N	3ix8	A	R137	N141	0.49	0.162	No
K65Q/Q69Y	1c27	A	Q130	Y134	0.22	0.233	No
K60E/E64Y	3ub9	A	E48	Y52	0.47	0.259	No
A73S/G77H	4toh	B	S126	H130	0.27	0.28	No
D127N/V131T	3bol	B	N190	T194	0.39	0.298	No
K19T/G23D	5df7	B	T168	D172	0.42	0.307	No
D72R/R76N	3ix8	A	R137	N141	0.5	0.314	No
E5S/I9K	4kn8	B	S26	K30	0.3	0.321	No
L39R/K43R	1qtx	B	R12	R16	0.47	0.335	No
D127S/V131H	4toh	B	S126	H130	0.27	0.37	No
T115S/R119D	4iqg	B	S37	D41	0.27	0.4	No

Mutation	Template	Chain	R1	R2	RMSD	ΔΔG	Clash
D61N/K65T	3bol	B	N190	T194	0.42	0.406	No
A73H/G77Y	1xh8	A	H142	Y146	0.43	0.428	No
K85N/D89T	3bol	B	N190	T194	0.35	0.428	No
T109H/G113S	1p7g	H	H87	S91	0.44	0.45	No
K65E/Q69Y	3ub9	A	E48	Y52	0.15	0.5	No
A41R/E45H	4xv8	A	R48	H52	0.21	0.506	No
K65D/Q69W	4ggo	A	D205	W209	0.23	0.544	No
D72N/R76T	3bol	B	N190	T194	0.33	0.647	No
K85H/D89N	4hmm	B	H177	N181	0.29	0.647	No
S44H/K48W	4enq	D	H251	W255	0.28	0.685	No
K85N/D89Y	3vlw	B	N375	Y379	0.3	0.711	No
N116Q/M120R	2x1c	B	Q196	R200	0.22	0.719	No
A41D/E45W	4ggo	A	D205	W209	0.17	0.768	No
T151N/T155H	3drz	B	N104	H108	0.45	0.774	No
A41H/E45W	4enq	D	H251	W255	0.32	0.786	No
E45Q/A49H	2gj3	A	Q29	H33	0.41	0.826	No
K65N/Q69Y	3vlw	B	N375	Y379	0.24	0.875	No
K85D/D89W	4ggo	A	D205	W209	0.27	0.876	No
D72R/R76W	1w35	A	R163	W167	0.32	0.91	No
K65H/Q69W	4enq	D	H251	W255	0.21	0.982	No
L39T/K43Q	3c9x	A	T109	Q113	0.41	0.984	No
D70Q/A74H	2gj3	A	Q29	H33	0.45	0.986	No

Mutation	Template	Chain	R1	R2	RMSD	ΔΔG	Clash
K19Q/Y25W	1g6i	A	Q311	W318	0.41	1.007	No
K85H/D89W	4enq	D	H251	W255	0.43	1.011	No
K60R/E64N	3ix8	A	R137	N141	0.2	1.013	No
K65H/Q69Y	1xh8	A	H142	Y146	0.17	1.061	No
K60H/E64N	4hmm	B	H177	N181	0.48	1.066	No
K85H/D89S	1p7g	H	H87	S91	0.37	1.066	No
K43N/D47T	3bol	B	N190	T194	0.39	1.12	No
P143S/K147D	4iqg	B	S37	D41	0.3	1.171	No
K65H/Q69N	4hmm	B	H177	N181	0.22	1.175	No
K65H/Q69K	1k3t	C	H349	K353	0.5	1.178	No
L39Q/K43R	2x1c	B	Q196	R200	0.46	1.191	No
K65H/Q69S	1p7g	H	H87	S91	0.44	1.199	No
L39R/K43W	1w35	A	R163	W167	0.49	1.204	No
K65N/Q69T	3bol	B	N190	T194	0.34	1.21	No
A41Q/E45Y	1c27	A	Q130	Y134	0.16	1.255	No
A73W/G77S	2eij	C	W146	S150	0.15	1.295	No
A41Q/E45H	2gj3	A	Q29	H33	0.41	1.304	No
N116K/M120H	2ifq	A	K39	H43	0.29	1.344	No
N116Y/M120T	4cae	C	Y88	T92	0.29	1.357	No
N116H/M120S	1p7g	H	H87	S91	0.38	1.395	No
A41H/E45Y	1xh8	A	H142	Y146	0.3	1.396	No
A41K/E45N	3lk4	W	K237	N241	0.29	1.424	No

Mutation	Template	Chain	R1	R2	RMSD	ΔΔG	Clash
E45H/A49S	1p7g	H	H87	S91	0.39	1.444	No
E5T/I9H	3vpc	C	T148	H152	0.26	1.465	No
K147H/T151S	1p7g	H	H87	S91	0.34	1.503	No
V75Q/L79R	2x1c	B	Q196	R200	0.34	1.516	No
N116H/E128H	5i14	A	H73	H85	0.36	1.528	No
K85D/D89N	4nac	B	D65	N69	0.44	1.53	No
V75W/L79Y	5std	C	W26	Y30	0.38	1.532	No
L39Q/K43Y	1c27	A	Q130	Y134	0.48	1.54	No
A41S/E45Y	3r3r	A	S159	Y163	0.44	1.543	No
V75E/L79Y	3ub9	A	E48	Y52	0.28	1.588	No
N116H/M120N	4hmm	B	H177	N181	0.25	1.589	No
E45N/A49E	4dno	B	N233	E237	0.48	1.595	No
V75Q/L79Y	1c27	A	Q130	Y134	0.31	1.61	No
A41S/E45H	4toh	B	S126	H130	0.16	1.615	No
N116Q/M120Y	1c27	A	Q130	Y134	0.21	1.616	No
E108T/A112S	3hh1	C	T43	S47	0.38	1.622	No
N116H/M120Y	1xh8	A	H142	Y146	0.25	1.632	No
F4N/R8T	3bol	B	N190	T194	0.43	1.634	No
L39T/K43H	3vpc	C	T148	H152	0.45	1.647	No
L39E/K43Y	3ub9	A	E48	Y52	0.43	1.653	No
E128N/N132T	3bol	B	N190	T194	0.35	1.672	No
Q69H/A73W	4enq	D	H251	W255	0.39	1.69	No

Mutation	Template	Chain	R1	R2	RMSD	ΔΔG	Clash
E5H/I9Y	1xh8	A	H142	Y146	0.26	1.697	No
K43H/D47N	4hmm	B	H177	N181	0.25	1.708	No
N116Y/M120Y	3i0z	A	Y292	Y296	0.49	1.71	No
A41H/E45S	1p7g	H	H87	S91	0.4	1.712	No
Q69H/A73Y	1xh8	A	H142	Y146	0.37	1.735	No
N116R/M120N	3ix8	A	R137	N141	0.45	1.736	No
L39T/K43R	3mux	A	T233	R237	0.23	1.742	No
E5N/I9Y	3vlw	B	N375	Y379	0.22	1.776	No
M120E/N132R	2jjb	C	E345	R371	0.29	1.801	No
L39K/K43N	3lk4	W	K237	N241	0.49	1.838	No
L39T/K43S	3hh1	C	T43	S47	0.39	1.878	No
L39W/K43S	2eij	C	W146	S150	0.46	1.932	No
D20S/Y24Q	3lum	C	S181	Q185	0.35	1.955	No
L39H/K43N	4hmm	B	H177	N181	0.44	1.976	No
A73H/G77S	1p7g	H	H87	S91	0.38	1.981	No
D70K/A74N	3lk4	W	K237	N241	0.37	2.02	No
L39Y/K43T	4cae	C	Y88	T92	0.46	2.03	No
V75N/L79Y	3vlw	B	N375	Y379	0.32	2.081	No
V75N/L79T	3bol	B	N190	T194	0.37	2.091	No
E108S/A112D	4iqg	B	S37	D41	0.38	2.143	No
Q69N/A73T	3bol	B	N190	T194	0.37	2.157	No
L39R/K43H	4xv8	A	R48	H52	0.46	2.182	No

Mutation	Template	Chain	R1	R2	RMSD	ΔΔG	Clash
N116S/M120Y	3r3r	A	S159	Y163	0.5	2.184	No
V71T/V75R	3mux	A	T233	R237	0.5	2.188	No
F67Q/V71Y	1c27	A	Q130	Y134	0.27	2.195	No
L66N/D70E	4dno	B	N233	E237	0.33	2.237	No
W126T/A130S	3hh1	C	T43	S47	0.2	2.237	No
V75K/L79H	2ifq	A	K39	H43	0.4	2.246	No
N116S/M120H	4toh	B	S126	H130	0.19	2.279	No
E5S/I9H	4toh	B	S126	H130	0.29	2.283	No
L39S/K43H	4toh	B	S126	H130	0.48	2.29	No
E45D/A49N	4nac	B	D65	N69	0.46	2.3	No
M120T/N132Q	2f7n	A	T72	Q112	0.29	2.308	No
M102E/M106E	3we5	B	E15	E19	0.21	2.311	No
V149N/F153Y	3vlw	B	N375	Y379	0.24	2.319	No
F67E/V71Y	3ub9	A	E48	Y52	0.2	2.34	No
M102K/M106E	3g9k	D	K212	E216	0.26	2.347	No
L39R/K43N	3ix8	A	R137	N141	0.25	2.354	No
C97T/N101H	3vpc	C	T148	H152	0.21	2.359	No
V75S/L79K	4kn8	B	S26	K30	0.32	2.383	No
V75W/L79T	1c5v	A	W237	T241	0.46	2.385	No
V75K/L79N	3lk4	W	K237	N241	0.14	2.411	No
N116S/M120K	4kn8	B	S26	K30	0.22	2.416	No
V71K/V75H	2ifq	A	K39	H43	0.29	2.483	No

Mutation	Template	Chain	R1	R2	RMSD	ΔΔG	Clash
V75Y/L79T	4cae	C	Y88	T92	0.3	2.489	No
V75H/L79Y	1xh8	A	H142	Y146	0.32	2.547	No
L91E/R95Q	2f57	B	E646	Q650	0.46	2.56	No
M106R/V111T	1vmg	A	R23	T28	0.39	2.572	No
L39S/K43D	4iqg	B	S37	D41	0.34	2.582	No
L39K/K43H	2ifq	A	K39	H43	0.39	2.599	No
V71W/V75Y	5std	C	W26	Y30	0.25	2.613	No
R145H/V149N	4hmm	B	H177	N181	0.28	2.627	No
V75S/L79H	4toh	B	S126	H130	0.31	2.631	No
V71K/I78R	4r0y	A	K827	R834	0.5	2.64	No
V149Q/F153R	2x1c	B	Q196	R200	0.26	2.641	No
V71Y/V75T	4cae	C	Y88	T92	0.27	2.646	No
V75W/L79S	2eij	C	W146	S150	0.32	2.652	No
V71N/V75T	3bol	B	N190	T194	0.34	2.671	No
R145S/V149Y	3r3r	A	S159	Y163	0.49	2.684	No
F67H/V71N	4hmm	B	H177	N181	0.2	2.69	No
V149D/F153W	4ggo	A	D205	W209	0.21	2.711	No
A41N/E45T	3bol	B	N190	T194	0.4	2.717	No
V75H/L79S	1p7g	H	H87	S91	0.39	2.74	No
F114R/L118S	3sxy	B	R166	S170	0.43	2.754	No
R145H/V149S	1p7g	H	H87	S91	0.45	2.772	No
I3T/L7Q	3c9x	A	T109	Q113	0.42	2.774	No

Mutation	Template	Chain	R1	R2	RMSD	ΔΔG	Clash
F114S/L118H	4o4t	A	S19	H23	0.45	2.788	No
V71H/V75N	4hmm	B	H177	N181	0.23	2.799	No
R145N/V149T	3bol	B	N190	T194	0.35	2.803	No
V75H/L79N	4hmm	B	H177	N181	0.36	2.824	No
S117W/L121T	1c5v	A	W237	T241	0.33	2.846	No
I100H/F104N	4hmm	B	H177	N181	0.24	2.874	No
I100Y/F104T	4cae	C	Y88	T92	0.21	2.88	No
F67K/V71N	3lk4	W	K237	N241	0.34	2.907	No
M6N/D10T	3bol	B	N190	T194	0.32	2.93	No
V71S/V75K	4kn8	B	S26	K30	0.24	2.947	No
S117N/L121E	4dno	B	N233	E237	0.39	2.971	No
M102E/V149T	2y81	A	E129	T232	0.49	2.975	No
D70S/A74D	4iqg	B	S37	D41	0.47	3.019	No
I150N/R154T	3bol	B	N190	T194	0.26	3.019	No
A74T/I78R	3mux	A	T233	R237	0.48	3.026	No
V71H/V75S	1p7g	H	H87	S91	0.41	3.039	No
I100N/F104T	3bol	B	N190	T194	0.37	3.062	No
S117Y/L121T	4cae	C	Y88	T92	0.26	3.084	No
F67N/V71E	4dno	B	N233	E237	0.47	3.085	No
R96T/I100H	3vpc	C	T148	H152	0.2	3.09	No
F67K/V71H	2ifq	A	K39	H43	0.26	3.092	No
F67N/V71T	3bol	B	N190	T194	0.31	3.092	No

Mutation	Template	Chain	R1	R2	RMSD	ΔΔG	Clash
I100S/F104K	4kn8	B	S26	K30	0.18	3.114	No
I3H/L7N	4hmm	B	H177	N181	0.47	3.124	No
V149Y/F153T	4cae	C	Y88	T92	0.32	3.125	No
V71W/V75T	1c5v	A	W237	T241	0.36	3.164	No
E62N/L66T	3bol	B	N190	T194	0.41	3.205	No
I100S/F104D	4iqg	B	S37	D41	0.48	3.225	No
V149N/F153T	3bol	B	N190	T194	0.39	3.249	No
A63N/F67E	4dno	B	N233	E237	0.4	3.265	No
V149H/F153N	4hmm	B	H177	N181	0.3	3.273	No
R96N/I100T	3bol	B	N190	T194	0.46	3.285	No
R96S/I100H	4toh	B	S126	H130	0.18	3.288	No
L118N/L133R	3g8d	B	N90	R111	0.33	3.321	No
L7N/E11H	3drz	B	N104	H108	0.46	3.323	No
M102N/M106H	3drz	B	N104	H108	0.45	3.346	No
I3S/L7K	4kn8	B	S26	K30	0.49	3.368	No
I100H/F104S	1p7g	H	H87	S91	0.39	3.427	No
L99E/V103Y	3ub9	A	E48	Y52	0.16	3.449	No
F67H/V71S	1p7g	H	H87	S91	0.43	3.46	No
I100S/F104H	4toh	B	S126	H130	0.18	3.46	No
L99K/V103H	2ifq	A	K39	H43	0.31	3.495	No
L99K/V103N	3lk4	W	K237	N241	0.29	3.504	No
V149S/F153H	4toh	B	S126	H130	0.16	3.523	No

Mutation	Template	Chain	R1	R2	RMSD	ΔΔG	Clash
V149S/F153K	4kn8	B	S26	K30	0.26	3.526	No
V71S/V75H	4toh	B	S126	H130	0.25	3.542	No
A146S/I150Y	3r3r	A	S159	Y163	0.42	3.556	No
S117H/L121S	1p7g	H	H87	S91	0.38	3.571	No
F67D/V71N	4nac	B	D65	N69	0.46	3.589	No
A129Y/L133T	4cae	C	Y88	T92	0.22	3.623	No
I3T/L7S	3hh1	C	T43	S47	0.42	3.648	No
A129T/L133H	3vpc	C	T148	H152	0.25	3.659	No
V149H/F153S	1p7g	H	H87	S91	0.43	3.689	No
A98S/M102H	4toh	B	S126	H130	0.19	3.713	No
L121D/L133E	2pqr	B	D101	E121	0.46	3.724	No
L121Q/L133E	3bve	B	Q88	E142	0.48	3.733	No
L99H/V103N	4hmm	B	H177	N181	0.27	3.735	No
L99N/V103T	3bol	B	N190	T194	0.4	3.735	No
F67S/V71D	4iqg	B	S37	D41	0.47	3.748	No
A42N/L46T	3bol	B	N190	T194	0.39	3.781	No
R95T/L99H	3vpc	C	T148	H152	0.31	3.804	No
A74S/I78H	4toh	B	S126	H130	0.29	3.805	No
A129S/L133K	4kn8	B	S26	K30	0.21	3.81	No
I3S/L7D	4iqg	B	S37	D41	0.45	3.823	No
L99N/V103E	4dno	B	N233	E237	0.41	3.837	No
A129S/L133H	4toh	B	S126	H130	0.33	3.845	No

Mutation	Template	Chain	R1	R2	RMSD	ΔΔG	Clash
V87E/L121N	4ev8	A	E571	N609	0.46	3.851	No
A146D/I150N	4nac	B	D65	N69	0.41	3.897	No
A146S/I150K	4kn8	B	S26	K30	0.16	4.019	No
L99S/V103K	4kn8	B	S26	K30	0.22	4.022	No
L99H/V103S	1p7g	H	H87	S91	0.43	4.024	No
A146S/I150H	4toh	B	S126	H130	0.27	4.029	No
L99S/V103H	4toh	B	S126	H130	0.16	4.043	No
L121S/L133Y	2h88	A	S497	Y537	0.31	4.102	No
L99S/V103Y	3r3r	A	S159	Y163	0.4	4.109	No
A129S/L133D	4iqg	B	S37	D41	0.46	4.225	No

Showing 1 to 272 of 272 entries

Table S7. The total number of contacts for the wild protein and their 50 mutants of the immunoglobulin (2YWY).

Proteus ID: UM6SQD. Available at <http://proteus.dcc.ufmg.br/result/id/UM6SQD>. The values represent the total atom pairs from different residues that attend to the cutoff patterns. Contacts were calculated using Arpeggio script tool (Jubb et al.*, 2017). P-value was calculated using T-Test function (parameters: paired and single tailed) from MS Excel software.*

*Definitions – wt: wild type; mt: mutant; hb: Hydrogen bond; whb: Weak hydrogen bond; i: Ionic interaction; a: Aromatic interaction; po: Polar interaction; wp: Weak polar interaction; hy: Hydrophobic interaction. *Each line represents a mutant compared to the unique wild type.*

#	Wt*							mt						
**Type**	**hb**	**whb**	**i**	**a**	**po**	**wp**	**hy**	**hb**	**whb**	**i**	**a**	**po**	**wp**	**hy**
**Contacts***	166	120	14	3	194	148	148	167	126	14	8	197	147	142
	166	120	14	3	194	148	148	166	122	14	0	195	149	139
	166	120	14	3	194	148	148	165	125	14	0	193	150	142
	166	120	14	3	194	148	148	166	122	14	3	195	157	141
	166	120	14	3	194	148	148	168	123	14	3	199	153	138
	166	120	14	3	194	148	148	167	121	14	3	199	153	146
	166	120	14	3	194	148	148	168	117	14	3	197	150	144
	166	120	14	3	194	148	148	166	120	14	3	194	153	145
	166	120	14	3	194	148	148	168	122	14	3	197	151	140
	166	120	14	3	194	148	148	167	118	14	10	197	154	150
	166	120	14	3	194	148	148	166	125	14	3	196	160	147
	166	120	14	3	194	148	148	166	124	14	3	199	158	142
	166	120	14	3	194	148	148	171	119	14	3	201	153	145
	166	120	14	3	194	148	148	168	121	14	3	200	153	147
	166	120	14	3	194	148	148	167	117	14	3	198	153	145
	166	120	14	3	194	148	148	167	115	14	3	197	155	148
	166	120	14	3	194	148	148	166	117	14	10	199	153	144
	166	120	14	3	194	148	148	168	116	14	3	200	151	146
	166	120	14	3	194	148	148	164	118	14	12	197	161	140
	166	120	14	3	194	148	148	166	120	14	3	198	157	146
	166	120	14	3	194	148	148	168	120	14	3	198	157	150
	166	120	14	3	194	148	148	169	121	14	3	201	157	146
	166	120	14	3	194	148	148	168	119	14	3	200	158	146
	166	120	14	3	194	148	148	170	125	14	3	200	154	148
	166	120	14	3	194	148	148	170	122	14	3	200	150	150
	166	120	14	3	194	148	148	166	122	14	3	196	155	150
	166	120	14	3	194	148	148	168	122	14	3	198	152	146
	166	120	14	3	194	148	148	166	122	14	3	198	154	146
	166	120	14	3	194	148	148	167	121	14	3	195	153	152
	166	120	14	3	194	148	148	168	123	14	3	196	157	147
	166	120	14	3	194	148	148	166	118	14	3	198	155	152
	166	120	14	3	194	148	148	166	120	14	3	196	154	148
	166	120	14	3	194	148	148	166	118	14	3	196	153	148

166	120	14	3	194	148	148	168	118	14	3	197	152	148
166	120	14	3	194	148	148	166	120	14	17	196	153	150
166	120	14	3	194	148	148	170	118	14	3	198	153	146
166	120	14	3	194	148	148	168	118	14	3	199	156	136
166	120	14	3	194	148	148	169	123	19	3	199	154	148
166	120	14	3	194	148	148	170	125	14	3	199	158	150
166	120	14	3	194	148	148	169	127	14	0	198	155	139
166	120	14	3	194	148	148	169	124	14	0	199	152	137
166	120	14	3	194	148	148	165	120	17	0	195	160	139
166	120	14	3	194	148	148	166	121	19	0	196	156	143
166	120	14	3	194	148	148	165	119	14	0	195	151	138
166	120	14	3	194	148	148	162	123	14	0	194	155	137
166	120	14	3	194	148	148	164	117	11	3	193	150	141
166	120	14	3	194	148	148	165	122	11	3	196	150	143
166	120	14	3	194	148	148	164	122	14	3	193	154	141
166	120	14	3	194	148	148	166	120	14	3	194	155	140
166	120	14	3	194	148	148	168	119	14	3	199	153	143
p-value							0,000200489	0,032347	0,215287	0,206438	1,27E-14	3,26E-19	2,28E-07

Table S8. The total number of contacts for the wild protein and their 61 mutants of the Protease (1LVB).

Proteus ID: N7Q9RZ. Available at <http://proteus.dcc.ufmg.br/result/id/ N7Q9RZ>. Contacts were calculated using Arpeggio script tool (Jubb et al., *2017). P-value was calculated using T-Test function (parameters: paired and single tailed) from MS Excel software.*

*Definitions – wt: wild type; mt: mutant; hb: Hydrogen bond; whb: Weak hydrogen bond; i: Ionic interaction; a: Aromatic interaction; po: Polar interaction; wp: Weak polar interaction; hy: Hydrophobic interaction. *Each line represents a mutant compared to the unique wild type.*

Protein	wt*							mt						
Type	hb	whb	i	a	po	wp	hy	hb	whb	i	a	po	wp	hy
Contacts*	303	251	17	44	347	275	335	305	251	17	44	351	279	332
	303	251	17	44	347	275	335	304	253	17	41	349	281	330
	303	251	17	44	347	275	335	303	249	17	44	351	288	331
	303	251	17	44	347	275	335	306	253	20	38	353	283	335
	303	251	17	44	347	275	335	305	253	17	38	352	283	336
	303	251	17	44	347	275	335	303	253	17	49	349	287	326
	303	251	17	44	347	275	335	305	253	17	44	349	283	331
	303	251	17	44	347	275	335	305	251	17	44	349	281	335
	303	251	17	44	347	275	335	303	253	17	44	349	285	323
	303	251	17	44	347	275	335	303	257	17	44	347	285	325
	303	251	17	44	347	275	335	305	253	17	44	349	286	326
	303	251	17	44	347	275	335	303	253	17	41	347	281	338
	303	251	17	44	347	275	335	303	251	17	41	346	288	327
	303	251	17	44	347	275	335	304	248	18	44	351	281	327
	303	251	17	44	347	275	335	303	253	17	52	351	280	326
	303	251	17	44	347	275	335	305	254	17	44	349	281	329
	303	251	17	44	347	275	335	305	255	17	44	350	284	330
	303	251	17	44	347	275	335	305	252	17	44	351	280	324
	303	251	17	44	347	275	335	303	251	17	44	349	275	327
	303	251	17	44	347	275	335	305	251	17	44	350	276	328
	303	251	17	44	347	275	335	303	252	17	44	351	281	328
	303	251	17	44	347	275	335	303	254	17	44	351	284	329
	303	251	17	44	347	275	335	306	253	20	44	352	282	328
	303	251	17	44	347	275	335	303	251	17	44	349	280	334
	303	251	17	44	347	275	335	305	253	17	44	351	282	330
	303	251	17	44	347	275	335	304	253	17	44	350	283	328
	303	251	17	44	347	275	335	303	255	17	44	351	283	330
	303	251	17	44	347	275	335	303	251	17	44	351	282	333
	303	251	17	44	347	275	335	303	251	17	58	351	284	332
	303	251	17	44	347	275	335	305	251	17	50	353	280	328
	303	251	17	44	347	275	335	305	253	17	44	351	282	335
	303	251	17	44	347	275	335	305	249	17	44	349	276	336

	303	251	17	44	347	275	335	301	250	17	44	344	281	336
	303	251	17	44	347	275	335	303	246	17	44	346	284	329
	303	251	17	44	347	275	335	305	251	17	48	352	283	343
	303	251	17	44	347	275	335	307	248	17	44	350	279	333
	303	251	17	44	347	275	335	305	252	17	49	352	278	328
	303	251	17	44	347	275	335	306	249	17	44	350	286	328
	303	251	17	44	347	275	335	303	251	17	44	349	281	329
	303	251	17	44	347	275	335	305	251	17	44	351	286	326
	303	251	17	44	347	275	335	303	251	17	44	351	285	329
	303	251	17	44	347	275	335	303	251	17	44	351	284	329
	303	251	17	44	347	275	335	305	251	17	44	352	283	331
	303	251	17	44	347	275	335	303	249	17	58	351	283	333
	303	251	17	44	347	275	335	303	249	17	39	351	282	332
	303	251	17	44	347	275	335	305	251	17	44	349	280	328
	303	251	17	44	347	275	335	304	250	17	44	349	281	330
	303	251	17	44	347	275	335	305	250	17	44	354	277	333
	303	251	17	44	347	275	335	306	253	20	44	352	279	334
	303	251	17	44	347	275	335	305	251	19	44	351	280	334
	303	251	17	44	347	275	335	306	251	17	44	354	284	337
	303	251	17	44	347	275	335	303	249	17	44	347	281	338
	303	251	17	44	347	275	335	303	251	17	44	353	285	337
	303	251	17	44	347	275	335	305	252	17	44	352	279	332
	303	251	17	44	347	275	335	305	253	17	44	351	285	337
	303	251	17	44	347	275	335	305	251	17	44	352	279	336
	303	251	17	44	347	275	335	303	252	17	44	351	286	335
	303	251	17	44	347	275	335	303	253	17	50	351	285	335
	303	251	17	44	347	275	335	305	251	17	48	349	283	337
	303	251	17	44	347	275	335	302	248	17	44	350	280	332
	303	251	17	44	347	275	335	305	255	17	53	355	285	324
p-value								3,4006 9E-10	0,020 989	0,016 363	0,043 059	1,15 E-19	1,08 E-27	8,41 E-10

Table S9. The total number of contacts for the wild protein and their 213 mutants of the Protease (1LGY).

Proteus ID: YTP3YC. Available at <http://proteus.dcc.ufmg.br/result/id/YTP3YC>. Contacts were calculated using Arpeggio script tool (Jubb et al., *2017). P-value was calculated using T-Test function (parameters: paired and single tailed) from MS Excel software.*

*Definitions – wt: wild type; mt: mutant; hb: Hydrogen bond; whb: Weak hydrogen bond; i: Ionic interaction; a: Aromatic interaction; po: Polar interaction; wp: Weak polar interaction; hy: Hydrophobic interaction. *Each line represents a mutant compared to the unique wild type.*

Protein	wt*							mt						
**Type**	**hb**	**whb**	**i**	**a**	**po**	**wp**	**hy**	**hb**	**whb**	**i**	**a**	**po**	**wp**	**hy**
**Contacts***	451	331	35	57	503	371	442	453	333	35	57	506	379	442
	451	331	35	57	503	371	442	453	333	35	57	506	382	444
	451	331	35	57	503	371	442	453	333	35	57	506	381	442
	451	331	35	57	503	371	442	449	326	35	57	503	377	441
	451	331	35	57	503	371	442	451	334	35	59	505	381	438
	451	331	35	57	503	371	442	452	336	35	57	505	383	440
	451	331	35	57	503	371	442	452	336	35	57	505	386	441
	451	331	35	57	503	371	442	450	336	35	64	505	381	443
	451	331	35	57	503	371	442	452	336	35	57	506	384	443
	451	331	35	57	503	371	442	453	332	35	57	506	379	436
	451	331	35	57	503	371	442	451	337	35	65	505	377	435
	451	331	35	57	503	371	442	451	336	35	67	505	382	440
	451	331	35	57	503	371	442	455	331	35	57	507	384	441
	451	331	35	57	503	371	442	455	333	35	57	510	376	440
	451	331	35	57	503	371	442	454	333	39	57	511	381	437
	451	331	35	57	503	371	442	447	331	31	57	506	378	442
	451	331	35	57	503	371	442	451	329	35	57	508	378	442
	451	331	35	57	503	371	442	453	326	38	57	506	380	440
	451	331	35	57	503	371	442	452	326	35	57	504	377	436
	451	331	35	57	503	371	442	451	326	35	57	504	376	434
	451	331	35	57	503	371	442	453	331	40	57	507	376	434
	451	331	35	57	503	371	442	450	328	40	57	505	377	437
	451	331	35	57	503	371	442	450	328	35	57	504	375	437
	451	331	35	57	503	371	442	451	328	35	57	504	376	435
	451	331	35	57	503	371	442	450	329	35	57	504	380	439
	451	331	35	57	503	371	442	452	328	37	57	507	377	436
	451	331	35	57	503	371	442	450	329	35	57	505	381	438
	451	331	35	57	503	371	442	452	326	35	57	505	374	434
	451	331	35	57	503	371	442	450	326	35	57	504	374	433
	451	331	35	57	503	371	442	450	328	38	57	504	375	433
	451	331	35	57	503	371	442	450	326	35	57	504	379	433
	451	331	35	57	503	371	442	452	327	35	57	504	378	435
	451	331	35	57	503	371	442	451	328	35	57	504	376	436

451	331	35	57	503	371	442	450	328	35	57	504	376	440
451	331	35	57	503	371	442	450	330	35	57	504	381	437
451	331	35	57	503	371	442	453	332	35	53	505	383	433
451	331	35	57	503	371	442	453	330	35	53	505	380	433
451	331	35	57	503	371	442	451	334	35	57	506	381	436
451	331	35	57	503	371	442	451	330	35	53	505	380	436
451	331	35	57	503	371	442	453	332	35	53	505	379	433
451	331	35	57	503	371	442	451	330	35	57	505	383	440
451	331	35	57	503	371	442	451	334	35	55	505	381	433
451	331	35	57	503	371	442	451	331	35	53	505	383	435
451	331	35	57	503	371	442	453	333	35	56	507	382	433
451	331	35	57	503	371	442	451	331	35	53	505	387	437
451	331	35	57	503	371	442	454	333	35	54	508	382	435
451	331	35	57	503	371	442	453	333	35	53	507	372	435
451	331	35	57	503	371	442	454	331	35	53	507	373	437
451	331	35	57	503	371	442	449	325	33	49	506	378	436
451	331	35	57	503	371	442	453	329	35	59	506	381	443
451	331	35	57	503	371	442	450	334	35	57	503	381	446
451	331	35	57	503	371	442	450	332	35	57	505	382	442
451	331	35	57	503	371	442	449	333	35	57	504	380	442
451	331	35	57	503	371	442	451	331	35	57	505	382	444
451	331	35	57	503	371	442	450	334	35	57	502	381	444
451	331	35	57	503	371	442	450	328	35	57	505	386	446
451	331	35	57	503	371	442	450	330	35	57	506	381	442
451	331	35	57	503	371	442	452	331	35	57	503	382	450
451	331	35	57	503	371	442	450	331	35	57	504	381	442
451	331	35	57	503	371	442	450	331	35	57	505	383	442
451	331	35	57	503	371	442	452	334	35	57	505	381	442
451	331	35	57	503	371	442	448	331	35	60	504	385	446
451	331	35	57	503	371	442	454	333	35	63	506	378	439
451	331	35	57	503	371	442	452	329	35	61	505	380	436
451	331	35	57	503	371	442	451	335	35	66	505	378	438
451	331	35	57	503	371	442	453	330	35	57	505	379	440
451	331	35	57	503	371	442	453	332	35	57	504	374	439
451	331	35	57	503	371	442	452	332	35	61	505	377	436
451	331	35	57	503	371	442	455	334	35	72	506	379	449
451	331	35	57	503	371	442	454	331	38	53	507	375	434
451	331	35	57	503	371	442	453	329	35	53	507	374	434
451	331	35	57	503	371	442	453	336	35	53	506	374	433
451	331	35	57	503	371	442	453	331	35	53	506	375	433
451	331	35	57	503	371	442	451	331	35	57	506	375	431
451	331	35	57	503	371	442	453	332	35	53	506	379	434
451	331	35	57	503	371	442	451	334	35	57	503	376	438

451	331	35	57	503	371	442	451	329	35	57	507	378	447
451	331	35	57	503	371	442	451	331	35	64	505	379	443
451	331	35	57	503	371	442	452	331	35	66	504	381	436
451	331	35	57	503	371	442	452	331	35	66	504	379	434
451	331	35	57	503	371	442	450	331	35	68	505	376	435
451	331	35	57	503	371	442	449	332	35	67	504	385	441
451	331	35	57	503	371	442	452	333	35	57	503	382	438
451	331	35	57	503	371	442	450	331	35	57	503	376	436
451	331	35	57	503	371	442	451	331	35	66	504	381	443
451	331	35	57	503	371	442	449	331	35	62	503	379	432
451	331	35	57	503	371	442	450	331	35	63	504	380	439
451	331	35	57	503	371	442	448	332	35	57	504	380	442
451	331	35	57	503	371	442	454	329	35	57	505	374	438
451	331	35	57	503	371	442	454	331	35	59	507	382	444
451	331	35	57	503	371	442	454	331	35	57	505	376	438
451	331	35	57	503	371	442	452	329	35	57	505	376	438
451	331	35	57	503	371	442	454	332	35	62	505	378	438
451	331	35	57	503	371	442	452	332	35	62	505	381	442
451	331	35	57	503	371	442	452	334	35	59	506	378	440
451	331	35	57	503	371	442	451	331	35	57	505	378	440
451	331	35	57	503	371	442	452	331	35	57	505	377	438
451	331	35	57	503	371	442	451	333	35	65	503	380	441
451	331	35	57	503	371	442	453	333	35	59	505	374	443
451	331	35	57	503	371	442	451	331	35	57	505	376	437
451	331	35	57	503	371	442	454	331	35	57	505	376	438
451	331	35	57	503	371	442	451	329	35	61	503	378	439
451	331	35	57	503	371	442	454	329	35	57	510	379	439
451	331	35	57	503	371	442	452	335	35	57	505	376	437
451	331	35	57	503	371	442	453	332	35	62	506	385	440
451	331	35	57	503	371	442	453	331	35	67	505	379	434
451	331	35	57	503	371	442	451	326	35	62	505	377	433
451	331	35	57	503	371	442	451	328	35	57	505	378	434
451	331	35	57	503	371	442	452	330	35	63	506	385	439
451	331	35	57	503	371	442	451	328	35	57	503	376	437
451	331	35	57	503	371	442	451	329	35	62	505	381	433
451	331	35	57	503	371	442	453	330	35	57	505	380	441
451	331	35	57	503	371	442	447	331	31	57	503	377	439
451	331	35	57	503	371	442	451	327	35	57	505	378	438
451	331	35	57	503	371	442	451	334	35	55	503	379	432
451	331	35	57	503	371	442	451	333	35	61	503	382	439
451	331	35	57	503	371	442	452	336	39	57	506	383	439
451	331	35	57	503	371	442	452	331	35	57	506	381	437
451	331	35	57	503	371	442	453	330	35	57	505	379	439

451	331	35	57	503	371	442	450	327	35	57	507	376	440
451	331	35	57	503	371	442	451	329	35	57	505	380	442
451	331	35	57	503	371	442	452	333	35	57	507	378	444
451	331	35	57	503	371	442	451	331	35	57	506	375	442
451	331	35	57	503	371	442	451	333	35	57	506	378	442
451	331	35	57	503	371	442	453	330	35	57	506	376	441
451	331	35	57	503	371	442	453	328	35	57	506	375	440
451	331	35	57	503	371	442	453	328	35	62	506	374	440
451	331	35	57	503	371	442	453	330	35	57	507	376	442
451	331	35	57	503	371	442	451	330	35	57	506	375	442
451	331	35	57	503	371	442	451	328	35	57	506	376	440
451	331	35	57	503	371	442	451	331	35	57	506	379	440
451	331	35	57	503	371	442	453	329	35	57	506	378	440
451	331	35	57	503	371	442	454	329	35	57	506	374	441
451	331	35	57	503	371	442	452	328	35	57	506	376	440
451	331	35	57	503	371	442	453	333	35	57	506	377	441
451	331	35	57	503	371	442	453	334	35	57	505	373	436
451	331	35	57	503	371	442	451	333	35	57	505	376	435
451	331	35	57	503	371	442	451	330	35	57	505	373	436
451	331	35	57	503	371	442	451	333	35	57	505	372	435
451	331	35	57	503	371	442	452	333	35	57	505	375	436
451	331	35	57	503	371	442	451	332	35	57	505	378	437
451	331	35	57	503	371	442	447	329	35	57	501	382	441
451	331	35	57	503	371	442	447	333	35	57	499	379	441
451	331	35	57	503	371	442	454	330	35	57	509	381	434
451	331	35	57	503	371	442	452	332	35	57	506	376	443
451	331	35	57	503	371	442	452	331	35	57	507	377	442
451	331	35	57	503	371	442	450	331	32	57	503	374	437
451	331	35	57	503	371	442	450	328	32	63	504	375	442
451	331	35	57	503	371	442	452	330	35	57	505	375	446
451	331	35	57	503	371	442	452	331	35	57	504	375	442
451	331	35	57	503	371	442	451	331	35	57	504	375	442
451	331	35	57	503	371	442	452	332	35	62	504	376	451
451	331	35	57	503	371	442	449	331	35	62	503	379	444
451	331	35	57	503	371	442	449	331	35	57	505	375	443
451	331	35	57	503	371	442	449	331	35	57	504	376	442
451	331	35	57	503	371	442	450	330	35	57	502	375	444
451	331	35	57	503	371	442	449	331	35	57	504	376	450
451	331	35	57	503	371	442	453	335	35	57	506	375	442
451	331	35	57	503	371	442	449	332	35	57	504	375	442
451	331	35	57	503	371	442	451	332	35	57	505	377	442
451	331	35	57	503	371	442	451	330	35	57	505	376	442
451	331	35	57	503	371	442	453	335	39	57	507	381	437

451	331	35	57	503	371	442	453	331	35	57	505	378	441
451	331	35	57	503	371	442	453	333	35	57	505	381	434
451	331	35	57	503	371	442	451	332	35	57	504	379	436
451	331	35	57	503	371	442	452	332	35	57	503	381	445
451	331	35	57	503	371	442	452	333	35	57	503	379	439
451	331	35	57	503	371	442	453	329	35	57	505	378	438
451	331	35	57	503	371	442	450	328	35	66	501	379	439
451	331	35	57	503	371	442	454	331	35	57	502	380	437
451	331	35	57	503	371	442	454	338	35	64	505	385	440
451	331	35	57	503	371	442	453	334	35	69	505	380	449
451	331	35	57	503	371	442	446	331	35	57	503	379	438
451	331	35	57	503	371	442	453	329	35	57	505	373	432
451	331	35	57	503	371	442	452	334	35	57	506	377	439
451	331	35	57	503	371	442	454	334	35	57	506	377	437
451	331	35	57	503	371	442	451	334	35	57	505	380	437
451	331	35	57	503	371	442	452	334	35	57	506	379	440
451	331	35	57	503	371	442	453	336	35	57	505	380	438
451	331	35	57	503	371	442	454	331	35	57	506	385	442
451	331	35	57	503	371	442	454	335	35	57	506	383	438
451	331	35	57	503	371	442	452	336	35	57	506	382	437
451	331	35	57	503	371	442	454	333	35	57	507	383	439
451	331	35	57	503	371	442	452	330	35	57	506	377	438
451	331	35	57	503	371	442	453	335	35	57	507	386	442
451	331	35	57	503	371	442	452	333	35	57	506	382	437
451	331	35	57	503	371	442	454	330	35	57	506	379	438
451	331	35	57	503	371	442	453	332	35	57	507	379	437
451	331	35	57	503	371	442	451	331	35	57	509	376	444
451	331	35	57	503	371	442	453	329	35	57	509	375	442
451	331	35	57	503	371	442	452	329	35	57	509	376	442
451	331	35	57	503	371	442	454	336	38	57	505	381	440
451	331	35	57	503	371	442	453	336	35	63	506	382	440
451	331	35	57	503	371	442	453	334	35	63	506	381	435
451	331	35	57	503	371	442	451	331	35	57	505	378	435
451	331	35	57	503	371	442	452	333	35	57	504	377	438
451	331	35	57	503	371	442	451	333	35	57	503	380	437
451	331	35	57	503	371	442	451	332	35	57	505	379	435
451	331	35	57	503	371	442	452	333	35	57	505	377	437
451	331	35	57	503	371	442	452	336	35	57	504	375	437
451	331	35	57	503	371	442	451	335	35	57	506	378	443
451	331	35	57	503	371	442	453	332	35	57	504	376	441
451	331	35	57	503	371	442	451	331	35	63	501	375	441
451	331	35	57	503	371	442	452	326	35	57	505	374	442
451	331	35	57	503	371	442	450	328	35	57	503	376	442

	451	331	35	57	503	371	442	450	328	35	57	505	379	442
	451	331	35	57	503	371	442	450	327	35	57	503	374	442
	451	331	35	57	503	371	442	450	327	35	57	505	375	444
	451	331	35	57	503	371	442	449	332	35	51	502	377	433
	451	331	35	57	503	371	442	450	333	35	51	500	370	435
	451	331	35	57	503	371	442	451	333	35	56	506	376	442
	451	331	35	57	503	371	442	453	330	35	57	504	372	443
	451	331	35	57	503	371	442	452	331	35	55	503	372	437
p-value								7,8934 3E-08	0,090 723	0,074 676	7,85 E-05	2,75 E-46	1,12 E-86	7,94 E-24

Table S10. The total number of contacts for the wild protein and their 344 mutants of the β-glucosidase (hydrolase; 1BGA).

Proteus ID: 4L8HLU. Available at <http://proteus.dcc.ufmg.br/result/id/4L8HLU>. Contacts were calculated using Arpeggio script tool (Jubb et al.*, 2017). P-value was calculated using T-Test function (parameters: paired and single tailed) from MS Excel software.*

*Definitions – wt: wild type; mt: mutant; hb: Hydrogen bond; whb: Weak hydrogen bond; i: Ionic interaction; a: Aromatic interaction; po: Polar interaction; wp: Weak polar interaction; hy: Hydrophobic interaction. *Each line represents a mutant compared to the unique wild type.*

Protein	wt*							mt						
Type	hb	whb	i	a	po	wp	hy	hb	whb	i	a	po	wp	hy
Contacts*	797	703	108	197	922	773	880	797	698	108	188	922	776	874
	797	703	108	197	922	773	880	800	703	108	188	925	783	874
	797	703	108	197	922	773	880	799	701	108	188	925	778	873
	797	703	108	197	922	773	880	799	706	108	188	926	782	875
	797	703	108	197	922	773	880	799	701	108	188	924	778	873
	797	703	108	197	922	773	880	797	704	108	194	924	779	873
	797	703	108	197	922	773	880	797	701	108	188	924	777	875
	797	703	108	197	922	773	880	797	700	108	196	924	782	877
	797	703	108	197	922	773	880	796	702	108	202	922	783	878
	797	703	108	197	922	773	880	797	703	108	197	923	780	881
	797	703	108	197	922	773	880	800	701	108	197	925	780	877
	797	703	108	197	922	773	880	800	701	108	197	924	779	878
	797	703	108	197	922	773	880	798	700	108	204	925	777	876
	797	703	108	197	922	773	880	797	702	105	197	923	775	880
	797	703	108	197	922	773	880	796	709	108	197	922	784	881
	797	703	108	197	922	773	880	796	704	108	197	924	778	880
	797	703	108	197	922	773	880	797	705	108	197	921	778	880
	797	703	108	197	922	773	880	797	704	108	197	923	776	880
	797	703	108	197	922	773	880	792	700	102	197	920	776	874
	797	703	108	197	922	773	880	794	698	100	202	920	780	874
	797	703	108	197	922	773	880	797	697	100	197	923	775	879
	797	703	108	197	922	773	880	797	702	105	197	925	784	882
	797	703	108	197	922	773	880	797	700	108	197	922	777	882
	797	703	108	197	922	773	880	797	698	108	197	922	773	880
	797	703	108	197	922	773	880	792	699	106	197	921	777	876
	797	703	108	197	922	773	880	795	699	106	197	923	779	881
	797	703	108	197	922	773	880	799	701	106	197	926	774	880
	797	703	108	197	922	773	880	797	701	106	197	925	777	882
	797	703	108	197	922	773	880	797	701	106	197	926	779	880
	797	703	108	197	922	773	880	799	702	106	197	927	775	880
	797	703	108	197	922	773	880	799	701	106	197	926	775	880
	797	703	108	197	922	773	880	799	701	106	197	925	775	880

797	703	108	197	922	773	880	797	701	106	197	925	776	880
797	703	108	197	922	773	880	797	701	106	197	927	777	880
797	703	108	197	922	773	880	799	701	106	204	926	776	886
797	703	108	197	922	773	880	796	701	108	201	923	779	876
797	703	108	197	922	773	880	797	701	108	197	924	777	878
797	703	108	197	922	773	880	801	700	109	197	928	780	877
797	703	108	197	922	773	880	797	700	108	197	925	778	877
797	703	108	197	922	773	880	797	703	108	197	924	782	873
797	703	108	197	922	773	880	799	703	108	192	925	778	876
797	703	108	197	922	773	880	797	704	108	192	924	775	875
797	703	108	197	922	773	880	800	705	108	192	924	777	877
797	703	108	197	922	773	880	798	704	108	194	924	780	880
797	703	108	197	922	773	880	799	704	108	192	924	778	873
797	703	108	197	922	773	880	799	705	108	192	924	780	876
797	703	108	197	922	773	880	797	700	108	194	924	783	877
797	703	108	197	922	773	880	797	697	108	189	922	777	865
797	703	108	197	922	773	880	799	698	108	197	924	776	866
797	703	108	197	922	773	880	795	697	108	193	924	776	866
797	703	108	197	922	773	880	797	697	108	203	926	786	875
797	703	108	197	922	773	880	799	699	108	189	926	777	870
797	703	108	197	922	773	880	797	696	108	189	924	776	868
797	703	108	197	922	773	880	800	699	108	189	926	777	868
797	703	108	197	922	773	880	797	698	108	189	924	779	865
797	703	108	197	922	773	880	797	698	108	189	925	781	869
797	703	108	197	922	773	880	797	698	108	198	924	781	873
797	703	108	197	922	773	880	797	698	108	214	922	782	884
797	703	108	197	922	773	880	797	698	108	191	924	783	874
797	703	108	197	922	773	880	799	705	108	193	924	775	872
797	703	108	197	922	773	880	799	708	108	193	925	779	869
797	703	108	197	922	773	880	799	699	108	199	929	784	877
797	703	108	197	922	773	880	799	704	108	199	925	784	870
797	703	108	197	922	773	880	799	702	108	201	927	778	881
797	703	108	197	922	773	880	797	704	108	204	925	783	877
797	703	108	197	922	773	880	800	704	108	197	927	777	878
797	703	108	197	922	773	880	800	703	112	197	925	778	878
797	703	108	197	922	773	880	797	706	108	197	924	785	877
797	703	108	197	922	773	880	795	711	108	198	921	779	878
797	703	108	197	922	773	880	797	712	108	198	921	779	879
797	703	108	197	922	773	880	796	705	101	189	919	772	880
797	703	108	197	922	773	880	797	702	106	194	921	780	880

797	703	108	197	922	773	880	798	702	105	194	921	781	879
797	703	108	197	922	773	880	797	703	105	194	922	774	878
797	703	108	197	922	773	880	798	704	105	200	922	781	882
797	703	108	197	922	773	880	798	699	109	196	922	776	877
797	703	108	197	922	773	880	797	701	105	197	921	783	881
797	703	108	197	922	773	880	797	701	108	197	925	779	879
797	703	108	197	922	773	880	797	699	108	197	926	776	876
797	703	108	197	922	773	880	799	700	108	197	925	778	875
797	703	108	197	922	773	880	797	699	108	197	925	778	875
797	703	108	197	922	773	880	797	697	108	197	925	778	879
797	703	108	197	922	773	880	799	701	108	197	925	780	876
797	703	108	197	922	773	880	796	707	108	190	923	778	878
797	703	108	197	922	773	880	798	705	108	184	923	781	878
797	703	108	197	922	773	880	797	703	108	187	921	777	881
797	703	108	197	922	773	880	801	704	108	197	925	784	874
797	703	108	197	922	773	880	797	699	108	197	924	780	873
797	703	108	197	922	773	880	801	703	108	197	925	780	871
797	703	108	197	922	773	880	799	701	108	197	924	776	873
797	703	108	197	922	773	880	797	696	110	197	925	781	873
797	703	108	197	922	773	880	797	701	108	197	924	781	877
797	703	108	197	922	773	880	796	707	105	194	922	783	876
797	703	108	197	922	773	880	799	705	108	197	925	782	883
797	703	108	197	922	773	880	797	706	108	197	924	782	873
797	703	108	197	922	773	880	799	705	108	197	923	779	874
797	703	108	197	922	773	880	798	700	108	197	924	782	874
797	703	108	197	922	773	880	800	704	108	197	925	785	872
797	703	108	197	922	773	880	798	704	108	197	924	779	871
797	703	108	197	922	773	880	800	707	108	197	926	783	873
797	703	108	197	922	773	880	797	698	108	197	923	782	872
797	703	108	197	922	773	880	797	701	108	209	923	779	872
797	703	108	197	922	773	880	797	699	108	197	922	782	874
797	703	108	197	922	773	880	799	701	105	197	925	780	876
797	703	108	197	922	773	880	795	700	105	197	925	776	872
797	703	108	197	922	773	880	799	705	108	197	926	777	872
797	703	108	197	922	773	880	797	702	105	197	926	778	876
797	703	108	197	922	773	880	795	703	105	197	925	781	874
797	703	108	197	922	773	880	797	702	105	199	925	776	872
797	703	108	197	922	773	880	796	704	108	197	924	777	875
797	703	108	197	922	773	880	799	705	110	197	924	775	882
797	703	108	197	922	773	880	796	701	108	197	922	777	877

797	703	108	197	922	773	880	800	700	108	197	928	781	872
797	703	108	197	922	773	880	799	706	108	197	925	780	876
797	703	108	197	922	773	880	798	699	108	197	927	782	875
797	703	108	197	922	773	880	801	708	108	207	925	783	876
797	703	108	197	922	773	880	799	706	108	197	924	782	869
797	703	108	197	922	773	880	801	703	108	197	924	775	871
797	703	108	197	922	773	880	797	702	108	197	924	776	869
797	703	108	197	922	773	880	792	702	102	197	919	776	876
797	703	108	197	922	773	880	799	700	108	197	927	777	875
797	703	108	197	922	773	880	799	707	108	194	925	776	871
797	703	108	197	922	773	880	797	707	108	194	924	775	873
797	703	108	197	922	773	880	797	706	108	204	922	776	871
797	703	108	197	922	773	880	797	705	108	196	925	775	869
797	703	108	197	922	773	880	799	707	108	194	925	777	871
797	703	108	197	922	773	880	797	702	108	197	922	777	871
797	703	108	197	922	773	880	799	706	108	197	924	775	880
797	703	108	197	922	773	880	799	705	108	197	924	776	873
797	703	108	197	922	773	880	798	703	108	197	924	779	872
797	703	108	197	922	773	880	799	705	108	197	925	777	875
797	703	108	197	922	773	880	798	705	108	197	924	777	875
797	703	108	197	922	773	880	799	708	108	197	924	780	873
797	703	108	197	922	773	880	797	704	108	197	924	776	873
797	703	108	197	922	773	880	798	705	108	197	925	775	874
797	703	108	197	922	773	880	797	704	108	197	924	775	868
797	703	108	197	922	773	880	797	706	108	197	925	779	868
797	703	108	197	922	773	880	797	706	108	197	924	780	870
797	703	108	197	922	773	880	797	710	108	197	924	782	876
797	703	108	197	922	773	880	797	701	104	189	921	777	878
797	703	108	197	922	773	880	799	703	105	189	922	784	877
797	703	108	197	922	773	880	804	702	108	200	930	781	880
797	703	108	197	922	773	880	797	703	108	197	923	784	872
797	703	108	197	922	773	880	800	703	108	197	924	785	873
797	703	108	197	922	773	880	797	700	108	197	924	783	871
797	703	108	197	922	773	880	797	703	108	207	924	780	871
797	703	108	197	922	773	880	797	699	108	197	924	784	874
797	703	108	197	922	773	880	800	699	108	205	924	782	873
797	703	108	197	922	773	880	799	701	108	197	924	776	879
797	703	108	197	922	773	880	799	701	108	197	924	779	879
797	703	108	197	922	773	880	797	703	108	197	924	777	879
797	703	108	197	922	773	880	797	703	110	197	922	777	887

797	703	108	197	922	773	880	796	704	111	197	921	783	881
797	703	108	197	922	773	880	794	702	108	197	921	775	879
797	703	108	197	922	773	880	794	700	108	197	921	775	879
797	703	108	197	922	773	880	797	703	108	197	924	776	880
797	703	108	197	922	773	880	797	701	108	197	922	781	877
797	703	108	197	922	773	880	799	701	108	197	922	781	876
797	703	108	197	922	773	880	799	701	112	197	925	782	881
797	703	108	197	922	773	880	797	699	110	197	927	777	880
797	703	108	197	922	773	880	799	704	110	204	923	777	874
797	703	108	197	922	773	880	800	699	108	197	926	778	882
797	703	108	197	922	773	880	801	702	108	197	926	786	885
797	703	108	197	922	773	880	792	703	102	197	919	773	876
797	703	108	197	922	773	880	796	705	108	197	923	784	872
797	703	108	197	922	773	880	794	704	103	207	923	788	875
797	703	108	197	922	773	880	795	700	104	197	920	777	876
797	703	108	197	922	773	880	796	704	108	197	922	777	876
797	703	108	197	922	773	880	793	702	107	197	922	780	876
797	703	108	197	922	773	880	795	702	104	197	922	778	876
797	703	108	197	922	773	880	792	701	104	201	921	776	875
797	703	108	197	922	773	880	795	701	104	197	922	781	877
797	703	108	197	922	773	880	794	703	104	197	922	783	879
797	703	108	197	922	773	880	797	699	108	197	925	778	884
797	703	108	197	922	773	880	796	697	108	197	924	776	880
797	703	108	197	922	773	880	799	701	108	202	923	778	880
797	703	108	197	922	773	880	797	700	108	202	923	780	880
797	703	108	197	922	773	880	797	701	108	197	925	776	880
797	703	108	197	922	773	880	796	700	108	197	924	776	880
797	703	108	197	922	773	880	797	700	108	197	924	778	880
797	703	108	197	922	773	880	798	699	108	197	925	782	884
797	703	108	197	922	773	880	797	700	108	197	922	776	882
797	703	108	197	922	773	880	797	699	108	197	924	777	885
797	703	108	197	922	773	880	797	699	108	197	921	776	887
797	703	108	197	922	773	880	798	703	108	192	924	782	874
797	703	108	197	922	773	880	800	706	104	201	921	782	879
797	703	108	197	922	773	880	797	702	104	197	921	775	879
797	703	108	197	922	773	880	795	705	108	197	925	778	877
797	703	108	197	922	773	880	799	703	108	197	925	775	879
797	703	108	197	922	773	880	801	704	108	197	922	781	879
797	703	108	197	922	773	880	797	702	108	197	922	778	874
797	703	108	197	922	773	880	797	700	108	202	925	779	881

797	703	108	197	922	773	880	799	701	108	203	924	781	875
797	703	108	197	922	773	880	797	706	108	200	924	782	873
797	703	108	197	922	773	880	797	702	108	202	925	780	878
797	703	108	197	922	773	880	799	704	108	197	924	784	876
797	703	108	197	922	773	880	797	703	108	197	924	781	875
797	703	108	197	922	773	880	798	704	108	202	925	783	879
797	703	108	197	922	773	880	799	704	108	197	924	780	875
797	703	108	197	922	773	880	797	704	108	202	924	778	875
797	703	108	197	922	773	880	799	702	108	197	924	776	877
797	703	108	197	922	773	880	798	706	108	197	923	783	877
797	703	108	197	922	773	880	795	703	108	197	922	778	876
797	703	108	197	922	773	880	795	705	108	197	922	778	875
797	703	108	197	922	773	880	794	698	106	197	919	778	876
797	703	108	197	922	773	880	798	699	106	197	923	778	875
797	703	108	197	922	773	880	796	699	106	197	921	779	874
797	703	108	197	922	773	880	794	701	106	197	921	776	876
797	703	108	197	922	773	880	796	698	108	197	922	778	875
797	703	108	197	922	773	880	800	701	108	201	925	788	874
797	703	108	197	922	773	880	797	701	108	203	925	784	873
797	703	108	197	922	773	880	798	699	108	197	925	779	875
797	703	108	197	922	773	880	797	700	108	197	924	782	872
797	703	108	197	922	773	880	797	701	108	205	924	782	872
797	703	108	197	922	773	880	798	707	109	197	923	782	872
797	703	108	197	922	773	880	800	700	111	197	926	781	881
797	703	108	197	922	773	880	800	705	110	197	925	781	872
797	703	108	197	922	773	880	798	704	108	197	925	778	870
797	703	108	197	922	773	880	800	703	113	202	925	781	876
797	703	108	197	922	773	880	799	706	108	197	925	782	876
797	703	108	197	922	773	880	799	709	108	197	924	782	874
797	703	108	197	922	773	880	797	702	108	197	924	780	872
797	703	108	197	922	773	880	799	704	108	197	925	781	881
797	703	108	197	922	773	880	798	702	108	197	923	777	877
797	703	108	197	922	773	880	798	702	108	197	924	777	881
797	703	108	197	922	773	880	797	704	108	197	924	776	875
797	703	108	197	922	773	880	797	705	108	197	924	779	871
797	703	108	197	922	773	880	797	705	108	197	924	780	874
797	703	108	197	922	773	880	797	702	108	197	924	782	879
797	703	108	197	922	773	880	797	703	108	197	924	779	882
797	703	108	197	922	773	880	797	704	107	197	923	774	879
797	703	108	197	922	773	880	797	699	105	197	922	776	876

797	703	108	197	922	773	880	796	699	108	197	922	772	874
797	703	108	197	922	773	880	799	701	111	197	924	777	880
797	703	108	197	922	773	880	798	701	110	197	924	773	880
797	703	108	197	922	773	880	799	703	108	197	924	774	882
797	703	108	197	922	773	880	797	701	108	197	922	774	884
797	703	108	197	922	773	880	797	700	108	197	924	778	887
797	703	108	197	922	773	880	799	702	110	197	925	773	882
797	703	108	197	922	773	880	797	704	108	197	924	774	880
797	703	108	197	922	773	880	797	701	108	197	924	778	883
797	703	108	197	922	773	880	798	702	108	197	924	773	883
797	703	108	197	922	773	880	799	701	108	197	924	776	886
797	703	108	197	922	773	880	797	704	108	197	924	779	885
797	703	108	197	922	773	880	799	704	108	197	928	779	879
797	703	108	197	922	773	880	797	704	108	197	929	778	880
797	703	108	197	922	773	880	799	706	108	197	928	780	880
797	703	108	197	922	773	880	798	705	108	197	924	781	877
797	703	108	197	922	773	880	797	703	108	197	924	782	875
797	703	108	197	922	773	880	797	706	108	197	924	783	876
797	703	108	197	922	773	880	799	704	108	197	924	785	875
797	703	108	197	922	773	880	799	700	108	197	924	778	877
797	703	108	197	922	773	880	797	700	108	201	925	781	880
797	703	108	197	922	773	880	801	698	108	201	924	776	876
797	703	108	197	922	773	880	796	700	108	202	924	779	875
797	703	108	197	922	773	880	799	701	108	207	924	779	876
797	703	108	197	922	773	880	797	701	108	208	925	781	877
797	703	108	197	922	773	880	800	701	108	190	924	775	881
797	703	108	197	922	773	880	799	702	108	200	925	776	879
797	703	108	197	922	773	880	799	704	108	197	924	775	877
797	703	108	197	922	773	880	797	698	108	197	925	777	876
797	703	108	197	922	773	880	797	704	108	201	924	776	879
797	703	108	197	922	773	880	797	701	108	199	926	776	876
797	703	108	197	922	773	880	797	699	108	197	925	779	875
797	703	108	197	922	773	880	797	700	108	200	924	781	877
797	703	108	197	922	773	880	797	701	108	202	924	775	876
797	703	108	197	922	773	880	797	699	108	201	924	779	881
797	703	108	197	922	773	880	797	702	108	197	924	776	875
797	703	108	197	922	773	880	797	703	108	204	925	778	872
797	703	108	197	922	773	880	798	705	111	197	923	781	877
797	703	108	197	922	773	880	794	704	103	197	922	779	875
797	703	108	197	922	773	880	793	705	103	197	920	778	878

797	703	108	197	922	773	880	792	702	103	197	921	779	875
797	703	108	197	922	773	880	794	700	103	197	921	786	880
797	703	108	197	922	773	880	799	708	108	197	929	782	874
797	703	108	197	922	773	880	801	702	108	197	928	781	874
797	703	108	197	922	773	880	798	703	108	197	922	781	873
797	703	108	197	922	773	880	797	702	108	197	924	776	879
797	703	108	197	922	773	880	797	698	108	197	924	779	868
797	703	108	197	922	773	880	799	702	108	197	924	781	869
797	703	108	197	922	773	880	798	700	108	197	925	782	872
797	703	108	197	922	773	880	797	700	108	197	924	779	870
797	703	108	197	922	773	880	798	701	108	197	924	781	871
797	703	108	197	922	773	880	798	699	108	197	924	777	870
797	703	108	197	922	773	880	798	700	108	199	925	789	872
797	703	108	197	922	773	880	797	699	108	197	922	780	871
797	703	108	197	922	773	880	797	698	108	197	924	783	867
797	703	108	197	922	773	880	797	700	108	197	924	782	868
797	703	108	197	922	773	880	799	698	108	197	924	783	871
797	703	108	197	922	773	880	797	700	108	197	924	784	874
797	703	108	197	922	773	880	801	704	108	202	924	786	879
797	703	108	197	922	773	880	801	699	112	196	925	776	879
797	703	108	197	922	773	880	798	698	110	201	923	778	871
797	703	108	197	922	773	880	796	698	110	198	924	776	873
797	703	108	197	922	773	880	798	698	108	188	924	777	875
797	703	108	197	922	773	880	796	698	108	188	923	772	872
797	703	108	197	922	773	880	796	698	108	195	924	774	869
797	703	108	197	922	773	880	796	698	110	188	923	776	874
797	703	108	197	922	773	880	800	700	108	200	925	779	879
797	703	108	197	922	773	880	797	709	108	197	921	785	875
797	703	108	197	922	773	880	795	704	108	197	921	775	875
797	703	108	197	922	773	880	797	706	108	207	922	779	881
797	703	108	197	922	773	880	795	710	108	207	921	777	873
797	703	108	197	922	773	880	795	706	108	197	921	779	875
797	703	108	197	922	773	880	795	704	108	209	921	778	878
797	703	108	197	922	773	880	794	705	109	193	918	779	877
797	703	108	197	922	773	880	796	707	109	193	922	780	876
797	703	108	197	922	773	880	793	708	108	193	920	776	877
797	703	108	197	922	773	880	799	709	108	198	925	781	881
797	703	108	197	922	773	880	796	711	105	193	920	777	877
797	703	108	197	922	773	880	796	704	107	193	921	780	879
797	703	108	197	922	773	880	794	704	105	193	920	777	877

	797	703	108	197	922	773	880	797	704	111	193	922	776	878
	797	703	108	197	922	773	880	794	705	107	193	920	782	877
	797	703	108	197	922	773	880	798	700	108	184	921	773	875
	797	703	108	197	922	773	880	798	698	108	184	921	775	874
	797	703	108	197	922	773	880	794	695	108	184	923	769	874
	797	703	108	197	922	773	880	797	700	108	184	922	772	877
	797	703	108	197	922	773	880	795	697	108	184	920	770	875
	797	703	108	197	922	773	880	799	698	108	184	924	768	876
	797	703	108	197	922	773	880	797	699	108	184	921	772	874
	797	703	108	197	922	773	880	795	697	108	184	921	774	874
	797	703	108	197	922	773	880	794	701	108	197	922	780	874
	797	703	108	197	922	773	880	794	699	108	197	922	780	876
	797	703	108	197	922	773	880	796	701	108	197	922	776	875
	797	703	108	197	922	773	880	796	703	108	197	925	780	879
	797	703	108	197	922	773	880	796	702	108	197	924	775	882
	797	703	108	197	922	773	880	799	703	108	196	924	781	876
	797	703	108	197	922	773	880	801	702	108	196	926	783	874
	797	703	108	197	922	773	880	796	702	108	196	924	779	873
	797	703	108	197	922	773	880	798	702	108	200	925	780	877
	797	703	108	197	922	773	880	798	699	108	196	926	778	874
	797	703	108	197	922	773	880	796	699	108	196	922	776	877
	797	703	108	197	922	773	880	796	702	108	199	924	780	877
	797	703	108	197	922	773	880	799	705	108	196	922	782	870
	797	703	108	197	922	773	880	799	708	108	196	922	780	871
	797	703	108	197	922	773	880	799	700	108	196	923	780	879
	797	703	108	197	922	773	880	796	702	108	196	922	776	869
	797	703	108	197	922	773	880	797	704	108	196	922	780	871
	797	703	108	197	922	773	880	798	701	108	196	922	778	873
	797	703	108	197	922	773	880	799	701	108	196	922	777	870
	797	703	108	197	922	773	880	798	699	108	196	923	775	871
	797	703	108	197	922	773	880	797	701	108	196	922	773	870
	797	703	108	197	922	773	880	799	702	108	195	925	782	879
p-value								9,94218E-06	7,22E-09	2,04E-05	0,096348	1,92E-47	2,6E-107	1,66E-52

Table S11. List of stabilizing mutations for the PDB: 2LZM collected from ProTherm.

Available at <https://www.iitm.ac.in/bioinfo/ProTherm>.

PDB_WILD	MUTATION
**2LZM**	I3L
**2LZM**	D20T
**2LZM**	I3L
**2LZM**	I3L
**2LZM**	A82P
**2LZM**	I3L
**2LZM**	D20S
**2LZM**	S38D
**2LZM**	N116D
**2LZM**	E22K
**2LZM**	T26S
**2LZM**	A82P
**2LZM**	E45A
**2LZM**	G113A
**2LZM**	G113A
**2LZM**	A82P
**2LZM**	A93P
**2LZM**	K16E
**2LZM**	G113A
**2LZM**	A82P
**2LZM**	A41V
**2LZM**	S44A
**2LZM**	N40D
**2LZM**	N40A
**2LZM**	S44A
**2LZM**	A41V
**2LZM**	A41V
**2LZM**	N144D
**2LZM**	G77A
**2LZM**	I3L
**2LZM**	N144D
**2LZM**	T151S
**2LZM**	V131A
**2LZM**	S44L
**2LZM**	F153L
**2LZM**	S44A
**2LZM**	N116D
**2LZM**	V131A
**2LZM**	S44M

**2LZM**	N40A
**2LZM**	S44I
**2LZM**	G113E
**2LZM**	A82P
**2LZM**	C54T
**2LZM**	T157R
**2LZM**	A41V
**2LZM**	F153L
**2LZM**	V131A
**2LZM**	A41D
**2LZM**	V131A
**2LZM**	S44Q
**2LZM**	V131A
**2LZM**	S44R
**2LZM**	S38D
**2LZM**	R154E
**2LZM**	S44K
**2LZM**	V131E
**2LZM**	F153L
**2LZM**	S44Y
**2LZM**	N116A
**2LZM**	E128A
**2LZM**	V131I
**2LZM**	V131A
**2LZM**	V131M
**2LZM**	K147E
**2LZM**	G30A
**2LZM**	E11H
**2LZM**	S44V
**2LZM**	V131L
**2LZM**	V131D
**2LZM**	A93T
**2LZM**	S44F
**2LZM**	S44W
**2LZM**	S44H
**2LZM**	A93P
**2LZM**	E45A
**2LZM**	S44T
**2LZM**	I3L

References

Dehouck,Y. *et al.* (2011) PoPMuSiC 2.1: a web server for the estimation of protein stability changes upon mutation and sequence optimality. *BMC Bioinformatics*, **12**, 151.

Hazes,B. and Dijkstra,B.W. (1988) Model building of disulfide bonds in proteins with known three-dimensional structure. *Protein Eng.*, **2**, 119–125.

Huang,L.-T. *et al.* (2007) iPTREE-STAB: interpretable decision tree based method for predicting protein stability changes upon mutations. *Bioinformatics*, **23**, 1292–1293.

Jubb,H.C. *et al.* (2017) Arpeggio: A Web Server for Calculating and Visualising Interatomic Interactions in Protein Structures. *J. Mol. Biol.*, **429**, 365–371.

Laimer,J. *et al.* (2015) MAESTRO--multi agent stability prediction upon point mutations. *BMC Bioinformatics*, **16**, 116.

Mariano,D. *et al.* (2019) A Computational Method to Propose Mutations in Enzymes Based on Structural Signature Variation (SSV). *Int J Mol Sci*, **20**.

Pandurangan,A.P. *et al.* (2017) SDM: a server for predicting effects of mutations on protein stability. *Nucleic Acids Res*, **45**, W229–W235.

Pires,D.E.V. *et al.* (2014a) DUET: a server for predicting effects of mutations on protein stability using an integrated computational approach. *Nucleic Acids Res.*, **42**, W314-319.

Pires,D.E.V. *et al.* (2014b) mCSM: predicting the effects of mutations in proteins using graph-based signatures. *Bioinformatics*, **30**, 335–342.

Sowdhamini,R. *et al.* (1989) Stereochemical modeling of disulfide bridges. Criteria for introduction into proteins by site-directed mutagenesis. *Protein Eng.*, **3**, 95–103.

Teng,S. *et al.* (2010) Sequence feature-based prediction of protein stability changes upon amino acid substitutions. *BMC Genomics*, **11**, S5.
